# Effects of an antimicrobial peptide on transport- and novel environment-induced stress in British Shorthair cats

**DOI:** 10.3389/fvets.2025.1724637

**Published:** 2026-02-05

**Authors:** Shaohao Chen, Miaomiao Zhang, Haoran Yan, Lan Ye, Qishan Xue, Yuansheng Wu, Kun Zhao, Yizhou Jiang, Qingxin Wang, Jiang Zhu, Yan Guo, Qingshen Liu, Baichuan Deng, Lingna Zhang

**Affiliations:** 1 Guangdong Provincial Key Laboratory of Animal Nutrition Control, College of Animal Science, South China Agricultural University, Guangzhou, China; 2 Hangzhou Yipusi Biotechnology Co., Ltd., Hangzhou, China

**Keywords:** antimicrobial peptides, cats, gut microbiota, metabolomics, stress, transcriptome

## Abstract

Antimicrobial peptides (AMPs) are natural short peptides with known immunomodulatory and anti-inflammatory properties. Their application in feline stress management have not been widely studied. This study aimed to evaluate the effects of dietary AMPs derived from *Saccharomyces cerevisiae* on cats exposed to transportation and novel environment. Twelve cats were randomly allocated to a control group or a group fed with AMPs. After pre-feeding for 2 weeks, all cats underwent a two-hour transportation and were subsequently housed individually in novel environment for 1 week. Behavioral observations, biochemical assays, gut microbiota analysis, transcriptomics, and metabolomics were performed. AMPs supplementation significantly increased nighttime sleep duration, reduced activity on transportation day, and lowered cat stress scores (CSS) during the first 3 days in the novel environment. In the open field test (OFT), AMPs reduced escape and pacing behaviors (*p* < 0.05). AMPs also significantly decreased serum levels of CRH, COR, SAA, IL-1β, and IL-6, while increasing IgG and Apo-A1 after recovery. Antioxidant capacity was also significantly improved by AMPs, as shown by the elevated GSH-Px and reduced MDA. Higher abundances of Bacteroides, *Prevotella*, and *Collinsella* (*p* < 0.05), and lower *Schaalia* (*p* < 0.05) were observed in the AMP group. Metabolomics revealed that AMPs primarily regulated the nutritional status and immune function of cats by affecting amino acid and lipid metabolism, thereby enhancing their stress resilience. Transcriptomic analysis indicated that AMPs significantly upregulated pathways related to immune function, cell signal transduction, inflammatory response, and lipid metabolism, while downregulating those associated with viral processes. Dietary supplementation of AMPs alleviates stress in cats, potentially by reducing inflammatory and oxidative stress, and modulating gut microbiota, as well as metabolic and immune pathways.

## 1 Introduction

With the improvement of living standards, and changes in population structure and attitudes toward pet ownership, companion animals, particularly dogs and cats are increasingly valued for the emotional support they provide to humans. In recent years, the domestic cat population has grown significantly, especially in developing countries such as China (1). This rise in cat ownership has introduced new challenges. Many routine aspects of pet care, including travel and changes in the environment, can inevitably induce stress in pets. These stressors may contribute to the development of abnormal behaviors (e.g., anxiety and depression) and a series of health consequences, including compromised immune function and gastrointestinal dysfunction in cats (2).

Stress response refers to the non-specific, systemic adaptive reactions of an organism when exposed to aversive stimuli from both internal and external environments. When animals perceive actual or potential threats, the stress response is activated, involving the stimulation of the sympathetic-adrenal-medullary axis (SAM) and the hypothalamic–pituitary–adrenal (HPA) axis (3), leading to a series of physiological and behavioral changes in cats. While moderate stress can confer immediate benefits—such as enhanced physical and cognitive performance, mobilization energy reserves, which are crucial for survival, excessive or chronic stress has many detrimental effects (e.g., impaired immune function, abnormal behavior, and endocrine disruptions) (4). Stress-induced alterations in the transcriptomic profile (i.e., changes in gene expression) have profound implications for an individual’s physiological state and behavior. Stress activates multiple signaling pathways (e.g., RIG-I-like receptor signaling pathway, ErbB signaling pathway, endocannabinoid signaling pathway), particularly those linked to stress-responsive and immune-related genes, resulting in significant modifications to metabolic and immune functions (5). Consequently, research into management and nutritional strategies for stress alleviation is essential for improving welfare in cats.

Antimicrobial peptides (AMPs) are small bioactive molecules typically composed of 5 to 50 amino acids. These short peptides exert antibacterial effects primarily by targeting bacterial cell membranes, disrupting membrane integrity, and rapidly killing bacteria (6, 7). Beyond their classical antimicrobial properties, AMPs modulate various biological processes and cellular structures, fulfilling functions such as the regulation of inflammatory responses (8, 9) and immune functions (10), mitigating oxidative stress, and promoting the growth of beneficial microbes (11). Recent studies have identified unique roles of AMPs in modulating emotion and behavior. Specifically, AMPs regulate the gut microbiota balance, which can interact with the central nervous system via the gut-brain axis, and enhance the expression of brain-derived neurotrophic factor (BDNF), thereby regulating host stress responses and behaviors (12–14).

*Saccharomyces cerevisiae* and its metabolites have been demonstrated in various animal models to enhance intestinal barrier function, modulate immune responses, and alleviate oxidative stress (15). Previous research has shown that *S. cerevisiae* can reverse gut dysfunction induced by acute stress in mice (16). These beneficial effects are associated with the ability of *S. cerevisiae* to produce a variety of bioactive substances. Building upon this, the present study aims to investigate the role of *S. cerevisiae*-derived AMP in a feline stress model, evaluating their potential to mitigate stress responses through the modulation of gut health and immune function. Existing research on the application of AMPs in pets remains limited. Our previous study on the stress-alleviating effects of AMPs in cats focused primarily on the modulation of gut microbiota and related metabolic pathways (17). By integrating behavioral assessments with a multi-omics approach—including metagenomics, metabolomics, and transcriptomics, the current study aimed to comprehensively elucidate the protective mechanisms through which AMPs mitigate stress and promote overall health in cats.

## 2 Materials and methods

### 2.1 Animal ethics

All experimental procedures were authorized by the Animal Care and Use Committee (approval numbers: 2024a020) and were performed following the guidelines of the Laboratory Animal Center at South China Agricultural University.

### 2.2 Treatment administration

#### 2.2.1 Animal and diet

Twelve British Shorthair blue cats (6 males and 6 females, approximately 5 years old) were randomly assigned to two treatment groups based on body weight (BW) and sex, including the control group (CON) and the group receiving basal diet supplemented with AMPs (AMP). The average BW of the CON group was 4.86 ± 0.33 kg, while that of the AMP group was 4.85 ± 0.36 kg, without significant difference between the two groups. All cats were orally administered one capsule per day. The CON group received an empty gelatin capsule, whereas the AMP group received an identical capsule containing 200 mg/kg BW of AMPs. The AMP product was isolated and extracted from *Saccharomyces cerevisiae* and provided by Hangzhou Yipusi Biotechnology Co., Ltd. (Hangzhou, China). The ingredients and analyzed chemical compositions of the basal diet are shown in Table 1.

**Table 1:** Ingredients and nutrient levels of the experimental diet.

Items	Experimental diet %
Ingredients (as-is basis, %)	
Fresh bone-in chicken	35
Fresh chicken	20
Dehydrated chicken	10
Chicken powder	9
Chicken fat	3
Fresh chicken hearts	3
Freeze-dried egg yolk	3
Potato powder	2.5
Tapioca starch	2.5
Freeze-dried chicken liver	2
Freeze-dried chicken breast	2
Purple sweet potato	3
Fresh chicken liver	2
Fish oil	2
Psyllium husk	0.2
Broccoli	0.1
Carrot	0.1
Apple	0.1
Cranberry	0.1
Blueberry	0.1
Brewer’s yeast powder	0.1
Spirulina powder	0.1
*Yucca schidigera* powder	0.1
Chemical compositions (DM basis, %)	
DM	93.80
OM	91.60
CP	45.97
EE	18.10
CF	3.70

#### 2.2.2 Experiment design

**Figure 1:**
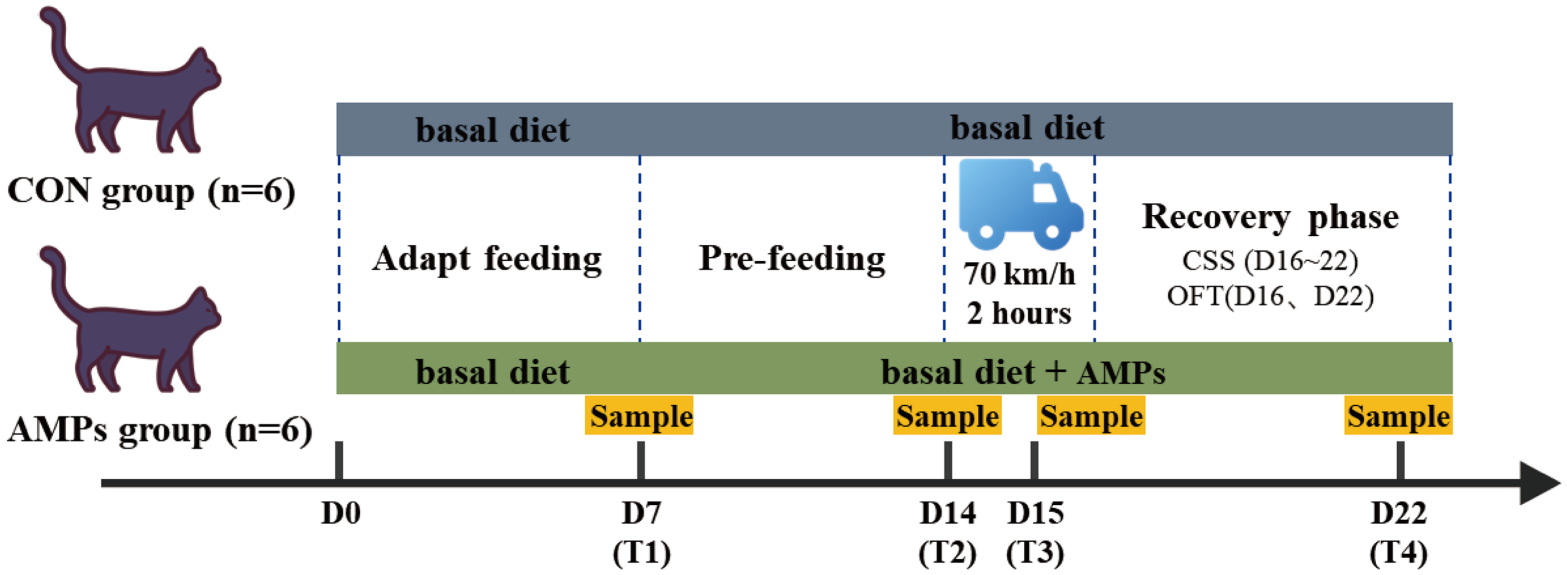
The sketch map of the study’s design. The control group (CON) was fed a basal diet (*n* = 6), and the group was fed a basal diet with antimicrobial peptides (AMP, *n* = 6). T1: End of diet transition period; T2: End of pre-feeding period (before transportation); T3: After transportation; T4: End of recovery period. CSS, cat stress score; OFT, open field test.

The experiment lasted 22 days and was divided into four phases, including an adaptation phase (D1-D7), a pre-feeding phase (D8-D14), a transportation phase (D15), and a recovery phase (D16-D22). Throughout the trial, each cat was individually housed in a cage (1.1 × 0.7 × 0.55 m) and fed once daily at 8:30 a.m. using an ad libitum feeding method, with free access to clean drinking water. Cats had 30-min play time outside the cage every day when they were brushed and allowed social interaction. A scratching pad and toy were also provided inside each cage. All cats had been routinely dewormed on a quarterly basis, and no medications (including antibiotics) were administered for 1 month period prior to the study (Figure 1).

### 2.3 Sample collection

#### 2.3.1 Average daily feed intake, fecal score, and body weight

Average daily feed intake (ADFI) and fecal scores were recorded. ADFI was measured prior to each feeding. Feces were classified on a five point scale, where score 1 represented dry, crumbly feces, score 2 represented dry feces with minimal moisture, score 3 represented normal, moist feces, score 4 represented soft feces, and score 5 represented diarrhea (18). Body weight was recorded for all cats before the start of the trial, and weekly throughout the experimental period.

#### 2.3.2 Monitoring of sleep quality and activity level

Due to limited equipment quantity, five cats were randomly selected from each group at the onset of the pre-feeding phase to wear the Actiwatch mini^®^ (CamNtech, UK) motion sensor. This device was used to monitor sleep duration, sleep quality, and activity levels throughout the trial period (19).

#### 2.3.3 Behavioral evaluation

Open field tests (OFT) were conducted on the first day following the transportation phase and at the end of the recovery phase to assess the stress levels and fear responses of the cats. The cats were individually placed in a 1.5 m × 2.5 m open, relatively quiet arena, where their behavior was recorded using two high-definition cameras positioned to capture a comprehensive view. The open field test lasted for 6 min, comprising 3 min for acclimatization to the arena and 3 min for behavioral recording. Between the testing of adjacent cats, the arena was sprayed with alcohol to eliminate residual odors from the previous animal, and the area was ventilated for 10 min to dissipate the alcohol before the next cat was tested.

During the recovery phase, stress-related behaviors were assessed daily using the Cat Stress Score (CSS), with each scoring session lasting approximately 30 min (20, 21). The first 10 min were set as an acclimatization period. Afterwards, the researcher stood approximately 30 cm from the cat’s cage and made eye contact for about 60 s without constantly staring at the cat. Following this, CSS assessment was conducted using the CSS reaction score sheet, which lasted for 1 min (20, 21). After the initial scoring, the cats were allowed 14 min to engage in other activity before repeating the 1-min CSS scoring. The final CSS score for each cat was the average of the two evaluations. All assessments were conducted after the morning feeding session. Two researchers jointly performed the scoring, recording a consensus CSS for each cat at every assessment point.

#### 2.3.4 Serum and fresh fecal sample collection

Blood samples (approximately 3 mL) were collected at four time points, at the end of the diet transition phase (T1), 1 h prior to transportation (T2), 1 h after transportation (T3), and at the end of the recovery phase (T4). The samples were collected into 10 mL vacuum blood collection tubes without anticoagulant, left to stand at room temperature for 30 min, and then centrifuged at 3,500 rpm for 15 min to obtain serum. The serum was aliquoted into centrifuge tubes and stored at −80 °C for subsequent analysis. Fresh fecal samples were collected within 10 min of defecation at T2, T3, and T4, and stored in cryovials at −80 °C for later testing.

#### 2.3.5 Peripheral blood mononuclear cell extraction

For peripheral blood mononuclear cell (PBMC) extraction, 3 mL of blood was collected in EDTA-coated tubes and stored at 4 °C. The PBMC samples were isolated using density gradient centrifugation (i.e., Ficoll method) as follows. A 15 mL sterile, non-enzymatic centrifuge tube was prepared with 5 mL of Ficoll-Paque lymphocyte separation solution. A second 15 mL non-enzymatic centrifuge tube was prepared with 3 mL of whole blood, which was diluted with 3 mL of 1 × PBS solution. The diluted blood was carefully layered onto the Ficoll-Paque solution in the first tube, ensuring clear separation. The sample was then centrifuged at 1,500 rpm for 30 min at room temperature, with acceleration and deceleration set to the slowest rates (no break, acceleration set at 1 and deceleration at 0). After centrifugation, the liquid was separated into four distinct layers, with the PBMC layer appearing as the middle thin white film. The upper layers were carefully aspirated, leaving approximately 1 mL of solution. The PBMC layer was carefully transferred into a new 15 mL non-enzymatic centrifuge tube. The cells were re-suspended in an appropriate volume of calcium- and magnesium-free Hank’s solution (D-Hank’s), mixed thoroughly, and centrifuged at 2,000 rpm for 10 min at 4 °C. The supernatant was discarded. This washing step was repeated twice, and the residual liquid was removed. The final cell pellet was re-suspended in 1 mL of TRIzol reagent, homogenized by pipetting, and transferred to RNase-free cryovial. The samples were then promptly shipped on dry ice to the testing platform for further analysis.

### 2.4 Data collection and analyses

#### 2.4.1 Sleep quality and activity level

The raw data downloaded from the Actiwatch mini® were analyzed using the accompanying Activity and Sleep Analysis 7 software. Sleep information from 00:00 to 08:00 was collected to assess the quality of nocturnal sleep, while data from 08:00 to 24:00 was used to evaluate the quality of daytime micro-sleep. Sleep quality metrics included total duration and frequency of sleep stages. Additionally, average activity levels and daily peak activity were recorded to assess overall activity levels.

#### 2.4.2 Behavioral evaluation

This study adapted open field test (OFT) to evaluate cat stress levels, a test widely used for stress determination in novel environment in different animals (22, 23). As shown in Table 2, behaviors related to stress and fear in cats were identified and categorized. A trained observer, blinded to treatment groups and experienced in behavioral coding and observation, analyzed the behavioral video recordings collected during the OFT. To assess inter-observer reliability, 33% of the samples (*n* = 4) were randomly selected and re-evaluated by a second trained observer. Based on the intragroup correlation coefficient (ICC) test, all six behavioral indicators demonstrated a high level of inter-observer agreement and reliability. Specifically, motionless (*R* = 0.891, *p* < 0.001), escape attempts (*R* = 0.890, *p* < 0.001), hiding behavior (*R* = 0.908, *p* < 0.001), exploration or play (*R* = 0.754, *p* < 0.01), pacing (*R* = 0.850, *p* < 0.01), and vocalization (*R* = 1) all exhibited strong consistency between raters.

**Table 2:** Behaviors evaluated in the open field test (23, 90).

Behavior type	Behavior definition	Measurement metric
Motionless	Maintaining a fixed body posture, such as standing, sitting, or lying down	Duration
Escape behavior	The cat crouches alertly near the arena exit, either staring at the exit or sniffing or using its paws to touch the exit	Duration
Hiding behavior	Curling up and hiding in a corner	Duration
Exploration or play	Sniffing, touching objects with the nose, mouth, or paws	Duration
Pacing	Repetitive, aimless walking or circling	Duration
Vocalization	The anxious vocalizations made by the cat after the start of the experiment	Frequency

#### 2.4.3 Serum biochemical analysis

Serum levels of catalase (CAT), total antioxidant capacity (T-AOC), glutathione peroxidase (GSH-Px), superoxide dismutase (SOD), and malondialdehyde (MDA) were measured using antioxidant assay kits (MEIMIAN, Jiangsu Meimian Industrial Co., Ltd., Yancheng, China). Additionally, serum concentrations of brain-derived neurotrophic factor (BDNF), adrenocorticotropic hormone (ACTH), corticotropin-releasing hormone (CRH), cortisol (COR), serum amyloid A (SAA), apolipoprotein A1 (Apo-A1), interleukins IL-1β, IL-6, IL-10, immunoglobulins IgA, IgG, IgM, tumor necrosis factor-alpha (TNF-α), and interferon-gamma (IFN-γ) were quantified using enzyme-linked immunosorbent assay (ELISA) kits (MEIMIAN, Jiangsu Meimian Industrial Co., Ltd., Yancheng, China). All procedures were conducted according to kit manual, and absorbance was measured using a multifunctional microplate reader for the determination of the concentrations of the analytes.

#### 2.4.4 Fecal short-chain fatty acids and branched-chain fatty acids

A total of 0.2 g of fecal sample was added to 1 mL of ultra-pure water, followed by vortexing for 5 min and ultrasonic treatment in an ice bath for 10 min. The mixture was then centrifuged at 13,000 rpm for 10 min at 4 °C. The entire supernatant was transferred to a new 2 mL centrifuge tube, to which 20 μL of 25% metaphosphoric acid and 0.25 g of anhydrous sodium sulfate were added. The mixture was vortexed for 1 min to facilitate acidification and salting-out. Subsequently, 1 mL of methyl tert-butyl ether (MTBE) was added under a fume hood, followed by vortexing for 5 min. The sample was then centrifuged at 13,000 rpm for 5 min at 4 °C, and the upper MTBE layer was carefully collected under a fume hood. Finally, the extract was filtered through a 0.22 μm microporous membrane and transferred into an autosampler vial with an insert for subsequent analysis. Quantitative analysis of short-chain fatty acids (SCFAs) and branched-chain fatty acids (BCFAs) was performed using a GCMS-QP 2020 system (Shimadzu, Kyoto, Japan). Instrumental parameters were optimized based on the methodology previously established in our laboratory (24).

#### 2.4.5 Metagenomic sequencing of fecal microbiota

The extraction of genomic deoxyribonucleic acid (DNA) was performed from the collected fecal samples. Purity was evaluated using a NanoDrop 2000 spectrophotometer, concentration was determined with a TBS-380 fluorometer, and DNA integrity was assessed by electrophoresis on a 1% agarose gel. The high-quality DNA was then fragmented to approximately 400 base pairs using a Covaris M220 focused-ultrasonicator. Construction of sequencing libraries was carried out using the NEXTFLEX Rapid DNA-Seq Kit, involving the ligation of adapters, magnetic bead-based removal of self-ligated adapter products, and subsequent polymerase chain reaction (PCR) amplification to enrich the target fragments. The amplified products were further purified using magnetic beads to obtain the final library. Sequencing was performed on an Illumina platform, where DNA molecules were clonally amplified on the flow cell via bridge PCR to form clusters. During sequencing, a modified DNA polymerase and four distinct fluorescently labeled deoxyribonucleotide triphosphates (dNTPs) were incorporated one at a time in a cyclic manner. After each incorporation, laser scanning was used to detect the specific fluorescent signal. The fluorescent and blocking groups were then removed to enable the next synthesis cycle. The complete sequences of DNA fragments were determined based on the cumulative fluorescent signals captured in each cycle.

#### 2.4.6 Untargeted metabolomics profiling and analysis

Serum metabolome were assessed using an untargeted approach via UPLC-MS/MS (Thermo Fisher Scientific, Waltham, MA, United States). Sample preparation and UPLC-MS/MS analysis were conducted as previously described (24). Raw data were processed using Compound Discoverer SP 3.3 software, and pathway enrichment analysis was performed in MetaboAnalyst 6.0,^1^ yielding visualized results for further interpretation.

#### 2.4.7 Transcriptome sequencing

The concentration and purity of RNA from PBMC were evaluated using a NanoDrop 2000 spectrophotometer. To proceed, the total RNA amount was required to be at least 1 μg, with a concentration ≥50 ng/μL, and an OD260/280 ratio between 1.8 and 2.2. Messenger RNA (mRNA) was then enriched using Oligo dT beads, followed by random fragmentation of the mRNA into approximately 300 bp segments. Complementary DNA (cDNA) synthesis was performed by reverse transcription using random primers, generating a single-stranded cDNA template, which was subsequently converted to double-stranded cDNA. The double-stranded cDNA was end-repaired, and an “A” base was added to the 3′ end for adapter ligation. After adapter ligation, the products were purified and size-selected, followed by PCR amplification to generate the final library. The library was quantified using the QuantiFluor dsDNA System, and the samples were pooled in appropriate ratios before being amplified on a cBot platform via bridge PCR to generate clusters. Finally, sequencing was performed on an Illumina platform (HiSeq Xten/NovaSeq6000).

### 2.5 Statistical data analysis

Data analysis was performed using SPSS 26.0 (IBM Corporation, Chicago, IL, United States) statistical software. One-way analysis of variance (ANOVA) was used to compare inter-group differences at consistent time points. For comparisons across different time points, a two-way repeated measures ANOVA with LSD-adjusted post-hoc tests was applied. Statistical significance was defined as *p* < 0.05, and trends were considered at *p* < 0.10. Results are presented as means ± standard errors. Graphical representations were generated using Origin 2022 (Origin Lab, Northampton City, MA, United States) and Adobe Illustrator 2024 (Adobe, San Jose, CA, United States).

## 3 Results

### 3.1 The effects of AMPs on body weight, average daily feed intake, and fecal score in cats

ADFI significantly decreased in both groups during the transportation phase, but the AMP group maintained a higher intake compared to the CON group (*p* < 0.05). By the end of the recovery period, ADFI in both groups had returned to pre-transportation baseline levels. Throughout the study, body weight remained consistent in both groups, and no significant changes in fecal condition were observed compared to baseline (Supplementary Figure S1).

### 3.2 The effects of AMPs on sleep time and activity level

We analyzed the sleep and activity patterns in the two groups (Supplementary Figure S2) and found that on the night after transportation, the AMP group exhibited longer sleep duration compared to the CON group (*p* < 0.05). Both groups showed a significant reduction in daytime sleep duration on the day of transportation relative to pre-transportation levels. However, the AMP group displayed a trend toward longer sleep duration than the CON group on the day of transportation (*p* < 0.10). After 1 week of housing in the new environment, sleep duration in both groups returned to baseline levels observed before transportation, although the AMP group still maintained longer daytime sleep compared to the CON group (*p* < 0.05).

Both groups exhibited a significant increase in activity on the day of transportation (*p* < 0.05); however, the CON group demonstrated higher activity levels than the AMP group (*p* < 0.05). After 1 week in the new housing environment, activity levels in both groups returned to pre-transportation baseline levels. Activity levels increased significantly during the transportation period in both groups, with the CON group exhibiting higher activity levels than the AMP group (*p* < 0.05). The CON group showed a significant decrease in activity during the first hour post-transportation compared to the same time period before transportation, whereas no significant decrease was observed in the AMP group. At 4 h post-transportation, activity levels were lower than pre-transportation levels in both groups (*p* < 0.05), with no significant difference between groups. The CON group exhibited a significant decrease in activity during the first 3 h post-transportation relative to pre-transportation levels (*p* < 0.05). In contrast, the AMP group showed a decrease only in the first hour (*p* < 0.05). During the second- and third-hours post-transportation, the AMP group maintained significantly higher activity levels than the CON group. A trend toward higher activity was also observed in the AMP group compared to the CON group during the first hour post-transportation (*p* < 0.10).

### 3.3 The impact of transport stress and novel environment on cat behavior in OFT

**Figure 2:**
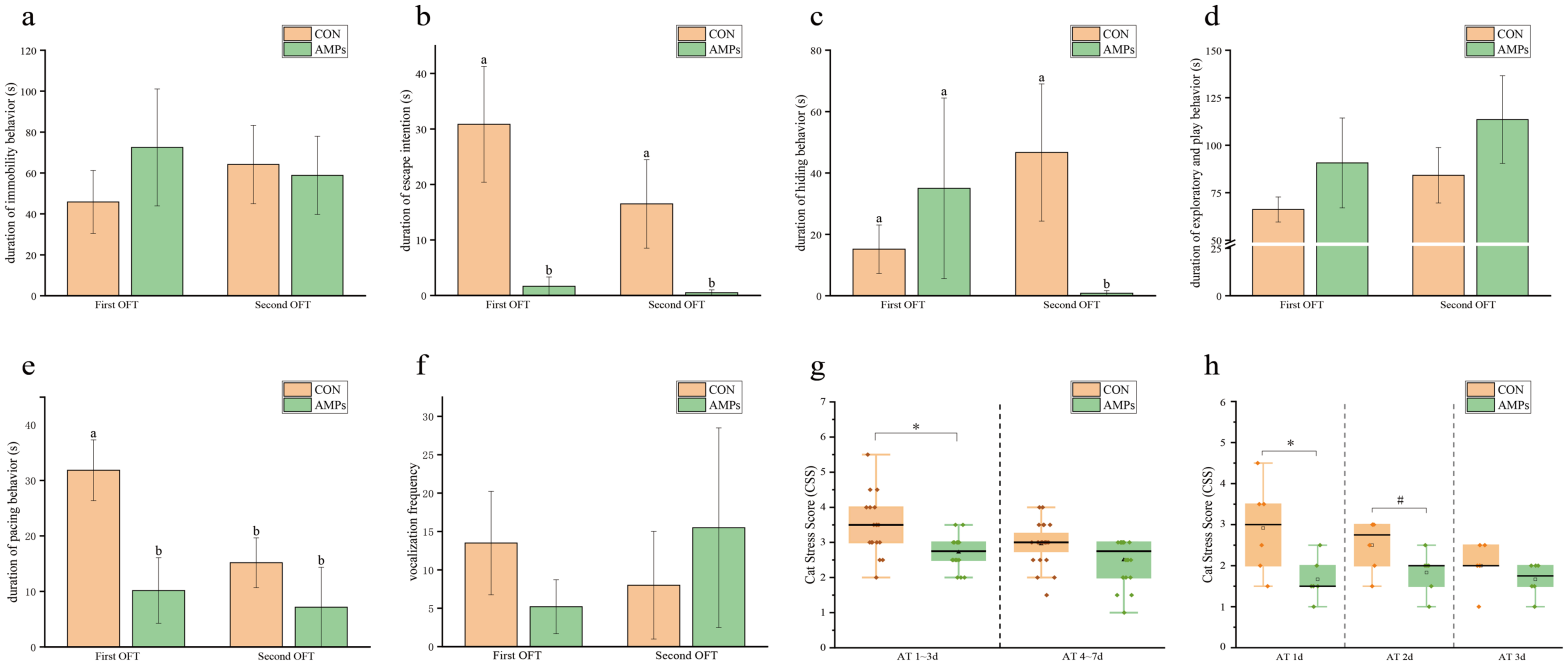
Effects of basal diet (CON) and antimicrobial peptides-supplemented diet (AMP) on open field test (OFT, **a–f**) and cat stress score (CSS, **g,h**). **(a)** Duration of immobility; **(b)** Duration of escape attempts; **(c)** Duration of hiding behavior; **(d)** Duration of exploratory behavior; **(e)** Duration of pacing; **(f)** Number of vocalizations; **(g)** Behavioral stress score during the recovery period (CSS); **(h)** CSS scores within 3 days after transportation. Data are presented as the mean ± SEM. Different lowercase letters (*) indicate statistically significant differences (*p* < 0.05), while the symbol (#) indicates a trend toward significance (*p* < 0.10). AT, right after transportation.

In OFT, no significant differences were observed between groups in terms of immobility duration, exploratory behavior, or vocalization frequency throughout the trial (Figures 2a,d,f). As shown in Figure 2b, the AMP group exhibited a lower escape intent during both OFT sessions compared to the CON group (*p* < 0.05). Regarding hiding duration, the CON group showed an increase in hiding time during the second test compared to the first test, although this difference was not statistically significant. In contrast, the AMP group demonstrated a significant reduction in hiding time, which was significantly lower than that of the CON group in the second test (Figure 2c). After 1 week of pre-feeding, the AMP group displayed less pacing behavior than the CON group during the first OFT session (*p* < 0.05, Figure 2e). No significant differences in vocalization frequency were detected between groups in both tests (Figure 2f).

Results of CSS indicated that the AMP group exhibited significantly lower stress levels compared to the CON group in the initial phase. Over time, the stress levels gradually improved in both groups, with the AMP group maintaining consistently lower scores than the CON group (Figure 2g). As shown in Figure 2h, on the first day after transportation, the CSS of the AMP group was significantly lower than that of the CON group. On the second day, the AMP group still showed a trend of lower CSS scores compared to the CON group (*p* < 0.10).

### 3.4 The effects of AMPs on the hematological parameters in cats

#### 3.4.1 Blood routine parameters

At T4, the white blood cells (WBC) count in the CON group increased significantly compared to T3, while no significant change was observed in the AMP group. As shown in Supplementary Figure S3, no significant difference in neutrophil (NEUT) counts was detected between the two groups at T3. Lymphocyte (LYM) levels decreased in both groups at T3 (*p* < 0.05) and returned to baseline levels (T2) by T4. Both red blood cell (RBC) count and hemoglobin (HGB) concentration showed parallel trends between two groups. Notably, the RBC count in the CON group at T2 exceeded the normal range, and both RBC and HGB levels decreased in both groups at T3. Significant difference was observed between T2 and T3 in the CON group for both parameters (*p* < 0.05), while HGB levels differed significantly between T3 and T4 in the AMP group (Supplementary Figure S3).

#### 3.4.2 BDNF and hormones of HPA axis

**Figure 3:**
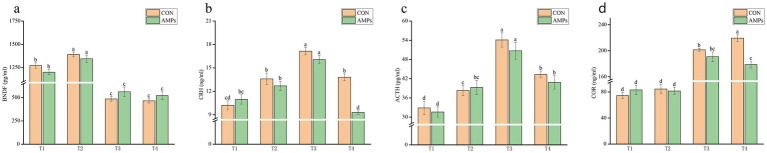
Effects of basal diet (CON) and antimicrobial peptides-supplemented diet (AMP) on BDNF and HPA axis hormones in the cats. **(a)** Brain-derived neurotrophic factor, BDNF; **(b)** Corticotropin-releasing hormone, CRH; **(c)** Adrenocorticotropic hormone, ACTH; **(d)** Cortisol, COR. Data are presented as the mean ± SEM. Different lowercase letters indicate statistically significant differences (*p* < 0.05). T1: End of diet transition period; T2: End of pre-feeding period (before transportation); T3: After transportation; T4: End of recovery period.

At T3, BDNF levels decreased significantly in both groups (*p* < 0.05; Figure 3a). CRH levels increased over time in both groups, with the CON group exhibiting a significant rise at T2 compared to T1. At T3, both groups showed a significant increase in CRH levels relative to other time points (*p* < 0.05). By T4, CRH levels had decreased significantly, with the AMP group demonstrated significantly lower CRH levels than the CON group (Figure 3b). Compared to T1, ACTH levels were elevated in both groups at T2 (*p* < 0.05), and ACTH levels at T3 were significantly higher than at T2 (*p* < 0.05). At T4, ACTH levels declined in both groups (*p* < 0.05, Figure 3c), with no significant difference between the groups. At T3, COR levels in the AMP group were numerically lower than those in the CON group. At T4, the CON group exhibited increase COR levels compared to T3 (*p* < 0.05), and these levels were significantly higher than those in the AMP group (*p* < 0.05, Figure 3d).

#### 3.4.3 Inflammatory cytokines and immunoglobulins

**Figure 4:**
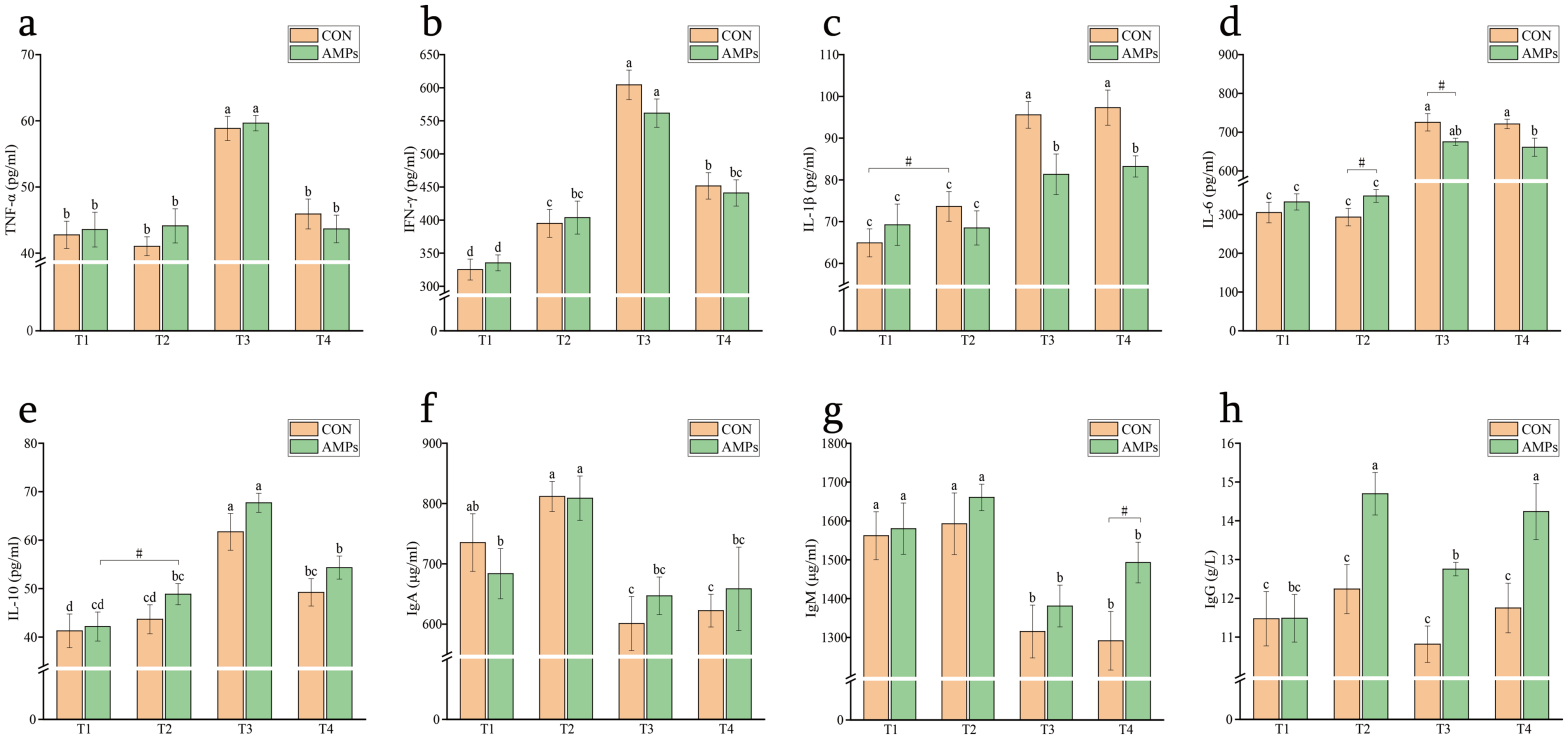
Effects of basal diet (CON) and antimicrobial peptides-supplemented diet (AMP) on serum inflammatory cytokines and immunoglobulins in the cats. **(a)** Tumor necrosis factor-α, TNF-α; **(b)** Interferon-γ, IFN-γ; **(c)** Interleukin-1β, IL-1β; **(d)** Interleukin-6, IL-6; **(e)** Interleukin-10, IL-10; **(f)** Immunoglobulin A, IgA; **(g)** Immunoglobulin M, IgM; **(h)** Immunoglobulin G, IgG. Data are presented as the mean ± SEM. Different lowercase letters indicate statistically significant differences (*p* < 0.05), while the symbol (#) indicates a trend toward significance (*p* < 0.10). T1: End of diet transition period; T2: End of pre-feeding period (before transportation); T3: After transportation; T4: End of recovery period.

At T3, both groups exhibited a significant increase in TNF-α levels, which subsequently decreased at T4 (*p* < 0.05), with no significant differences between the two groups (Figure 4a). IFN-γ levels increased gradually from T1 to T3 (*p* < 0.05) and declined significantly at T4; however, the CON group still exhibited significantly higher levels at T4 compared to T2 (Figure 4b). As shown in Figures 4c,d, levels of IL-1β and IL-6 increased significantly at T3. The AMP group exhibited lower IL-1β concentrations than the CON group at T3 (*p* < 0.05), and a trend toward lower IL-6 levels was also observed in the AMP group compared to the CON group (*p* < 0.10). Figure 4e shows that IL-10 levels in the AMP group tended to increase at T2 (*p* < 0.10), and both groups showed a significant elevation at T3, followed by a decrease at T4 (*p* < 0.05).

As shown in Figure 4f, IgA levels in the AMP group at T2 were higher than those at T1 (*p* < 0.05), while both groups showed a decrease at T3 (*p* < 0.05). For IgM, a significant decrease was observed at T3 compared to both T1 and T2 (*p* < 0.05), while at T4, the IgM levels in the AMP group showed gradual recovery, with a trend toward higher concentrations compared to the CON group (*p* < 0.10, Figure 4g). As shown in Figure 4h, the AMP group exhibited higher IgG levels than the CON group at T2 (*p* < 0.01). Although IgG levels in the AMP group declined significantly at T3, they remained higher than those in the CON group (*p* < 0.05). By T4, IgG levels in both groups had increased and returned to the baseline levels observed at T2; however, the AMP group still maintained higher IgG concentrations compared to the CON group (*p* < 0.01).

#### 3.4.4 Other serum hormones and antioxidant parameters

**Figure 5:**
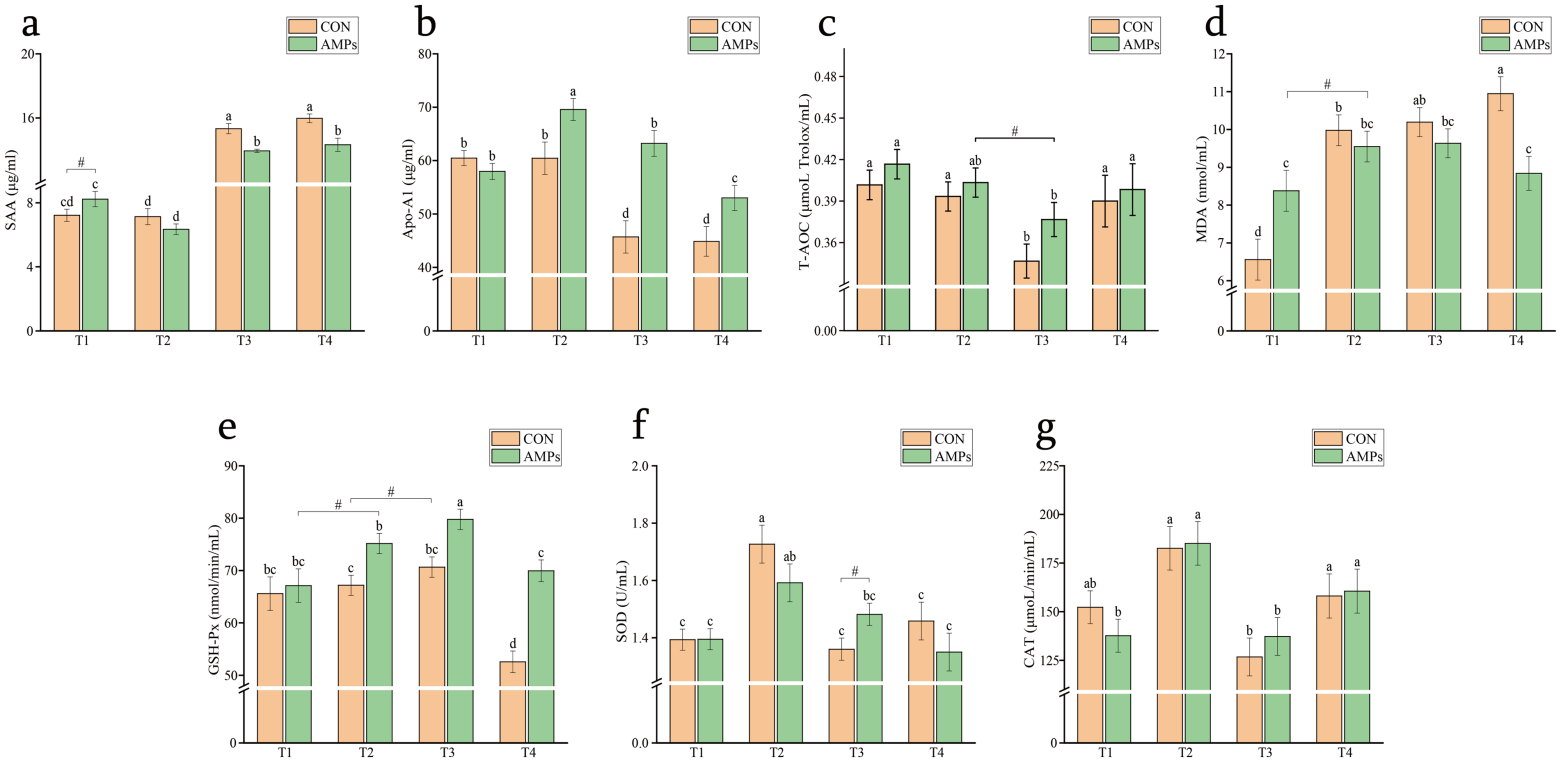
Effects of basal diet (CON) and antimicrobial peptides-supplemented diet (AMP) on serum hormone levels and antioxidant capacity in the cats. **(a)** Serum amyloid A, SAA; **(b)** Apolipoprotein A1, ApoA1; **(c)** Total antioxidant capacity, T-AOC; **(d)** Malondialdehyde, MDA; **(e)** Glutathione peroxidase, GSH-Px; **(f)** Superoxide dismutase, SOD; **(g)** Catalase, CAT. Data are presented as the mean ± SEM. Different lowercase letters indicate statistically significant differences (*p* < 0.05), while the symbol (#) indicates a trend toward significance (*p* < 0.10). T1: End of diet transition period; T2: End of pre-feeding period (before transportation); T3: After transportation; T4: End of recovery period.

At T2, SAA levels in the AMP group were lower than those at T1 (*p* < 0.05). Following transportation, SAA levels in both groups increased significantly, but the AMP group exhibited significantly lower level than the CON group. At T4, SAA levels remained higher in both groups compared to T1 and T2, but levels in the AMP group were maintained lower than those in the CON group (*p* < 0.05, Figure 5a). As shown in Figure 5b, Apo-A1 levels in the AMP group at T2 were higher than those at T1 and in the CON group (*p* < 0.05). At T3, Apo-A1 levels decreased significantly in both groups (*p* < 0.05), yet the CON group remained lower than those in the AMP group (*p* < 0.01).

T-AOC levels were lower in the CON group at T3 compared to T2 (*p* < 0.01), but recovered at T4 to levels comparable to T2 (*p* < 0.05). In contrast, T-AOC levels in the AMP group showed a slight decreasing trend at T3 (*p* < 0.10), but fully recovered to T2 levels by T4 (Figure 5c). MDA levels in the CON group increased at T2 (*p* < 0.01) and remained elevated thereafter. The AMP group showed significantly higher MDA levels than the CON group at T1, followed by a further increasing trend at T2 (*p* < 0.10). By T4, MDA levels in the AMP group had decreased and were significantly lower than those in the CON group (Figure 5d). As shown in Figure 5e, GSH-Px levels in both groups increased steadily, with a significant rise observed in the AMP group at T3, while both groups showed a decrease at T4 compared to T2 and T3 (*p* < 0.05). SOD and CAT levels increased in both groups at T2 (Figures 5f,g). At T3, SOD levels remained stable in the AMP group, whereas levels decreased in the CON group and CAT levels decreased in both groups compared to T2 (*p* < 0.05). By T4, CAT levels in both groups had increased significantly (Figures 5f,g).

### 3.5 Effects of antimicrobial peptides on the fecal microbiota composition in cats

#### 3.5.1 Fecal short-chain fatty acids and branched-chain fatty acids

As shown in Supplementary Figure S4, acetate and propionate levels decreased in both groups over time during the experimental period, with no significant differences observed between groups. At T2 and T3, butyrate levels in the CON group were significantly higher than those in the AMP group. After 1 week of acclimation, butyric acid in the CON group decreased compared to T2 (*p* < 0.05), while no change was observed between in the AMP group at T4.

Isobutyric acid levels increased in both groups in T4 compared to T2 and T3, but no significant differences were observed between the treatment groups. No significant differences in isovaleric acid or valeric acid levels were observed between the two groups throughout the experiment.

#### 3.5.2 Alpha and beta diversity analysis of fecal microbiota

As shown in Supplementary Figure S5, there are no significant differences in the Shannon and Simpson indices between the two groups at any time point. However, the Chao1 index of both groups decreased at T3 (*p* < 0.05), while the goods_coverage index increased at T3 (*p* < 0.05). PCoA results revealed no clear separation between the groups, indicating that the microbial compositions in the different fecal samples were similar.

#### 3.5.3 The composition and abundance of the fecal microbiota

**Figure 6:**
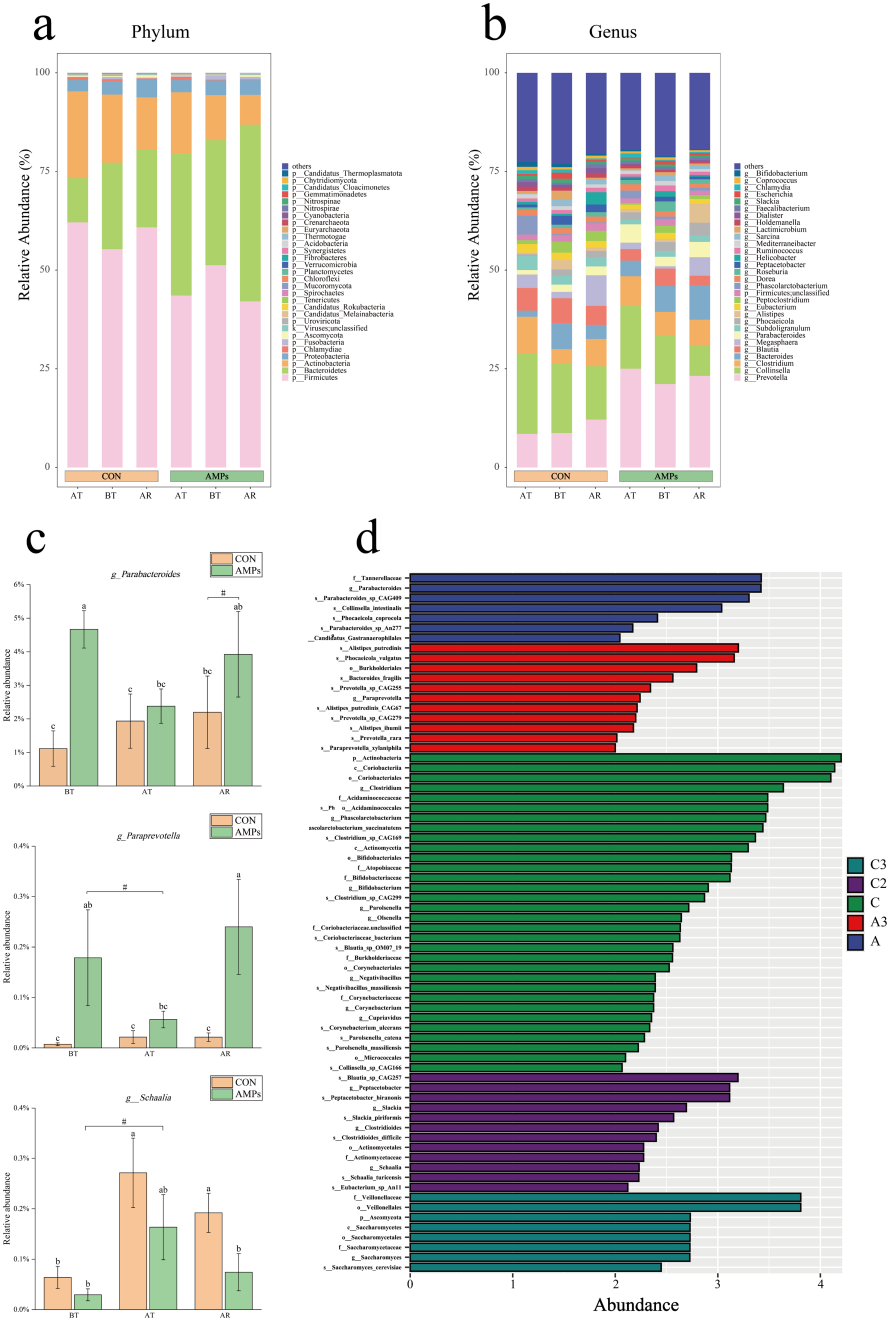
Effects of basal diet (CON) and antimicrobial peptides-supplemented diet (AMP) on gut microbiome in the cats. **(a)** histogram of the relative abundance at the phylum level; **(b)** histogram of the relative abundance at the genus level; **(c)** relative abundances *parabacteroides, paraprevotella, schaalia*; **(d)** LEFSe analysis between the CON and AMP group. Data are presented as the mean ± SEM. Different lowercase letters indicate statistically significant differences (*p* < 0.05), while the symbol (#) indicates a trend toward significance (*p* < 0.10). T2: End of pre-feeding period (before transportation); T3: After transportation; T4: End of recovery period.

The composition of the gut microbiota in both experimental groups was analyzed at three time points, as shown in Figures 6a,b. At the phylum level, the dominant phyla in both groups across all time points were Firmicutes, Bacteroidetes, Actinobacteria, and Proteobacteria (Figure 6a). At the genus level, the dominant taxa included *Prevotella*, *Collinsella*, *Clostridium*, *Bacteroides*, *Blautia*, and *Megasphaera* (Figure 6b).

As shown in Figure 6c, at the genus level, the relative abundance of *Prevotellaceae* and *Parabacteroides* was higher in the AMP group throughout the study period. Both genera were more abundant in AMP group than in the CON group at T2 (*p* < 0.05). *Parabacteroides* was more abundant in the AMP group (*p* < 0.05), while *Prevotellaceae* showed a trend of higher abundance in the AMP group (*p* < 0.10) at T2. The relative abundance of *Schaalia* was consistently higher in the CON group than in the AMP group throughout the study, with an increase observed at T4 (*p* < 0.05, Figure 6c).

Further LEfSe analysis was conducted to identify microbial differences between the two groups at different time points. As shown in Figure 6d, *S-Collinsella-intestinalis*, *g-Parabacteroides*, *g-Paraprevotella*, and *f-Tannerellaceae* were enriched in the AMP group, while *c-Coriobacteriia*, *g-Clostridium*, *p-Actinobacteria*, *g-Schaalia*, *g-Negativibacillus*, and *s-Clostridioides* difficile were enriched in the CON group.

### 3.6 Effects of antimicrobial peptides on blood transcriptomic profiles in cats

#### 3.6.1 Gene expression analysis

Venn diagram analysis identified 27 differentially expressed genes (DEGs) across all time points compared to the CON group. Transcriptomic analysis comparing the AMP and CON groups at each time points revealed a total of 2,315 DEGs. Specifically, 534, 341, 672, and 768 DEGs were detected in the comparisons of A1 vs. C1 (T1), A2 vs. C2 (T2), A3 vs. C3 (T3), and A4 vs. C4 (T4), respectively. Furthermore, a total of 1,159 genes were upregulated and 1,156 genes were downregulated in the AMP group compared to the CON group across the different time points (Supplementary Figure S6).

#### 3.6.2 Gene ontology enrichment analysis

**Figure 7:**
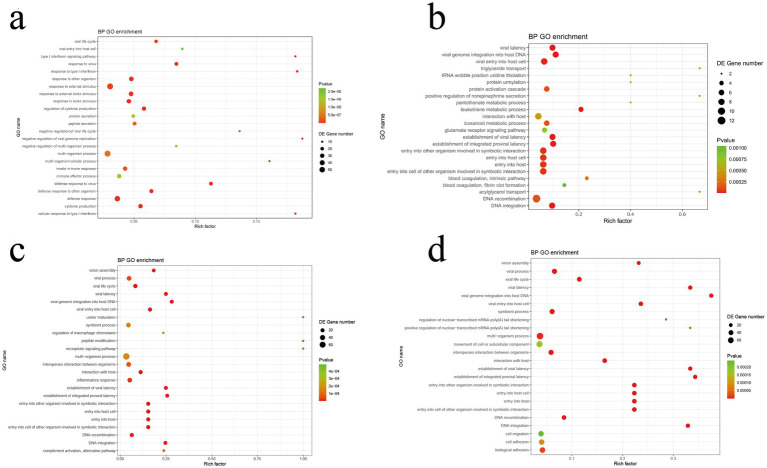
GO enrichment analysis of differentially expressed genes (DEGs) at various time points. **(a)** A1 vs. C1, End of diet transition period (T1); **(b)** A2 vs. C2, End of pre-feeding period (T2); **(c)** A3 vs. C3, After transportation (T3); **(d)** A4 vs. C4, End of recovery period (T4).

Gene ontology (GO) enrichment analysis was conducted to elucidate the biological functions of the DEGs. At T1, compared to the CON group, the AMP group exhibited significant upregulation (*p* < 0.01) of biological processes (BPs) related to immune effector processes, and defense responses to viruses and other pathogens. In contrast, processes such as biological adhesion, triglyceride transport, viral entry into host cells, and cell differentiation were significantly downregulated (*p* < 0.01; Figure 7a). After the pre-feeding period (T2), significantly enriched and upregulated BPs included anterograde trans-synaptic signaling, leukotriene metabolic process, eicosanoid metabolic process, and protein activation cascade. Conversely, virus-related processes such as viral process, viral life cycle, B cell homeostatic proliferation, DNA recombination, and acylglycerol transport were markedly downregulated (*p* < 0.01; Figure 7b).

After transportation (T3), the experimental group showed significant upregulation of BPs associated with complement activation (classical and alternative pathways), inflammatory response, muscle adaptation, and regulation of Hippo signaling (*p* < 0.01). Meanwhile, necroptotic signaling pathway, vasculature development, multi-organism processes, symbiotic interactions, and viral particle assembly were significantly downregulated (*p* < 0.01; Figure 7c). During the recovery phase (T4), GO enrichment analysis indicated significant upregulation (*p* < 0.01) of BPs involved in cell signaling, response to external stimuli, and metabolism of fatty acid derivatives. Conversely, processes associated with multi-organism interactions and virus-related life processes, including assembly, life cycle, latency, and host cell invasion, and DNA metabolic processes were significantly downregulated (*p* < 0.01; Figure 7d).

#### 3.6.3 KEGG pathway enrichment analysis

KEGG pathway enrichment analysis was conducted to further explore the biological pathways linked to the DEGs. As shown in Supplementary Figure S7, compared with the CON group, a total of 285 genes were enriched across 150 pathways at T1, 108 genes in 77 pathways at T2, 223 genes in 146 pathways at T3, and 197 genes in 128 pathways at T4. At T1, significantly enriched and upregulated pathways included the RIG-I-like receptor signaling pathway, tryptophan metabolism, and cytokine–cytokine receptor interaction (*p* < 0.05). At T2, pathways showing significant upregulation were primarily associated with pantothenate and CoA biosynthesis, complement and coagulation cascades, arachidonic acid metabolism, and ECM–receptor interaction (*p* < 0.05). Conversely, the adipocytokine signaling pathway, fat digestion and absorption, and neuroactive ligand–receptor interaction were significantly downregulated (*p* < 0.05). At T3, the complement and coagulation cascades, arachidonic acid metabolism, and purine metabolism pathways were significantly upregulated in the AMP group (*p <* 0.05). Conversely, the TGF-β signaling pathway, TNF signaling pathway, viral carcinogenesis, and Toll-like receptor (TLR) signaling pathway were significantly downregulated (*p* < 0.05). At T4, significant upregulation was observed in the ErbB signaling pathway, dopaminergic synapse, and retrograde endocannabinoid signaling pathway.

### 3.7 Effects of AMPs on feline metabolites under transport-induced and novel environment-induced stress

#### 3.7.1 Fecal metabolite profiles in cats

Principal component analysis (PCA) and partial least squares discriminant analysis (PLS-DA) were performed on fecal metabolites collected during the transportation phase. PCA results indicated no clear separation between the CON and AMP groups at any time points. In contrast, PLS-DA score plots revealed distinct separations between the two groups across all time points, suggesting the presence of differential metabolites. Further analysis identified 72 differentially expressed metabolites in fecal samples collected in T2. Among these, 50 metabolites were significantly upregulated and 22 were downregulated in the AMP group compared to the CON group. At T3, 56 differential metabolites were detected, of which 26 significantly upregulated and 30 significantly downregulated in the AMP group. At T4, a total of 147 differentially expressed metabolites were identified between the groups, with 90 significantly upregulated and 57 significantly downregulated in the AMP group.

**Figure 8:**
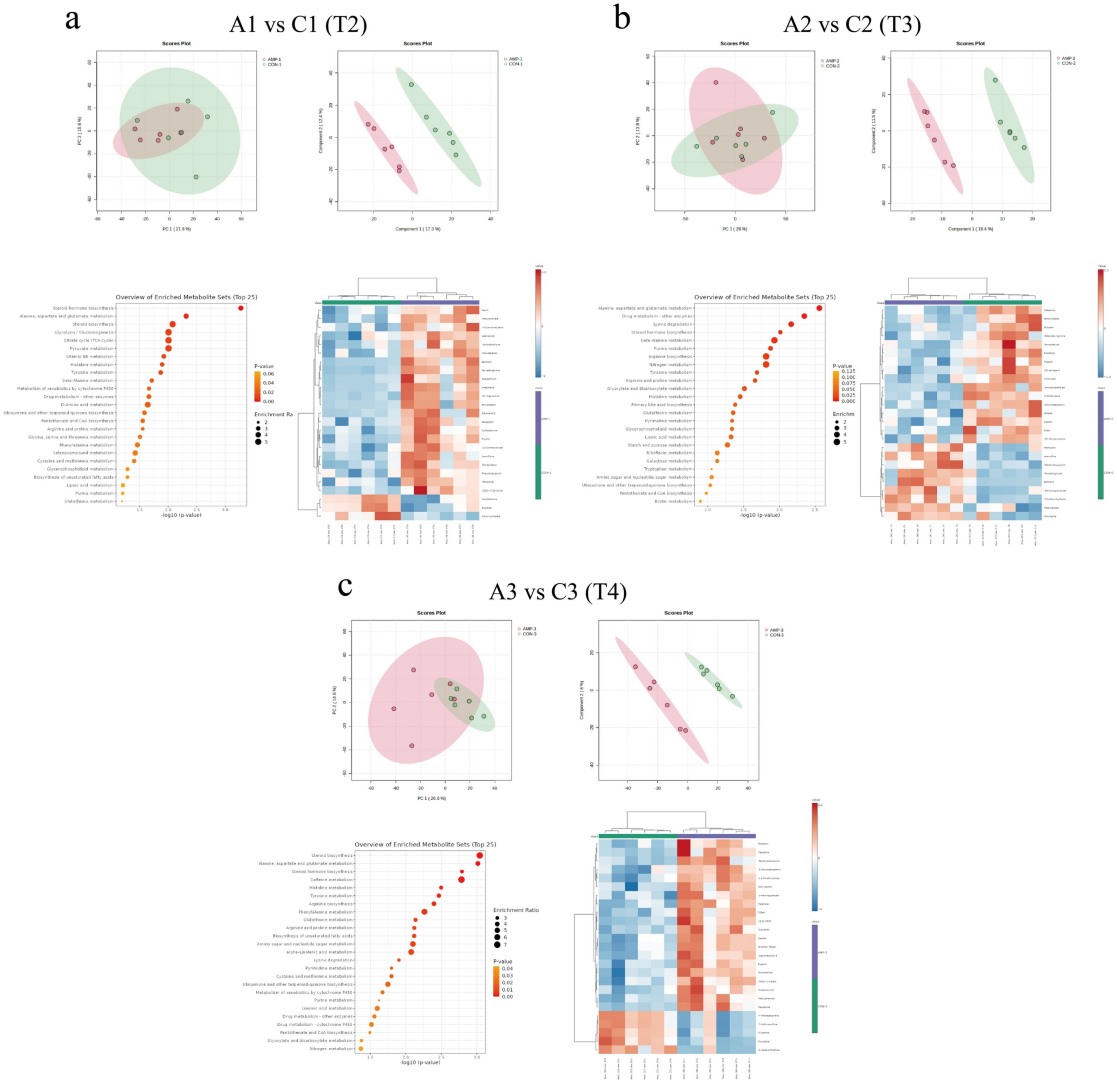
Effects of basal diet (CON) and antimicrobial peptides-supplemented diet (AMP) on fecal metabolites in cats. **(a)** Fecal metabolites prior to transportation [T2: end of pre-feeding period (before transportation)]; **(b)** Fecal metabolites after transportation (T3: after transportation); **(c)** Fecal metabolites after the recovery period (T4: end of recovery period). The panels include principal component analysis (PCA), partial least squares discriminant analysis (PLS-DA), the top 25 differentially expressed metabolites, and KEGG pathway enrichment bubble plots. Red and blue boxes represent positive and negative correlations.

KEGG pathway enrichment analysis revealed that AMPs primarily modulated pathways involved in amino acid metabolism, including arginine and proline metabolism, alanine, aspartate and glutamate metabolism, glycine, serine and threonine metabolism, and histidine metabolism, as well as steroid hormone biosynthesis, riboflavin metabolism, glycolysis/gluconeogenesis, and arachidonic acid metabolism at T2 (Figure 8a). After transportation (T3), AMPs additionally influenced linoleic acid metabolism, biotin metabolism, tyrosine metabolism, tryptophan metabolism, and alpha-linolenic acid metabolism (Figure 8b). By T4, the differential metabolites were predominantly enriched in pathways related to amino acid metabolism (e.g., arginine and proline metabolism, alanine, aspartate, and glutamate metabolism, histidine metabolism, and tyrosine metabolism), linoleic acid metabolism, riboflavin metabolism, and alpha-linolenic acid metabolism (Figure 8c).

#### 3.7.2 Effects of AMPs on serum metabolites in cats

**Figure 9:**
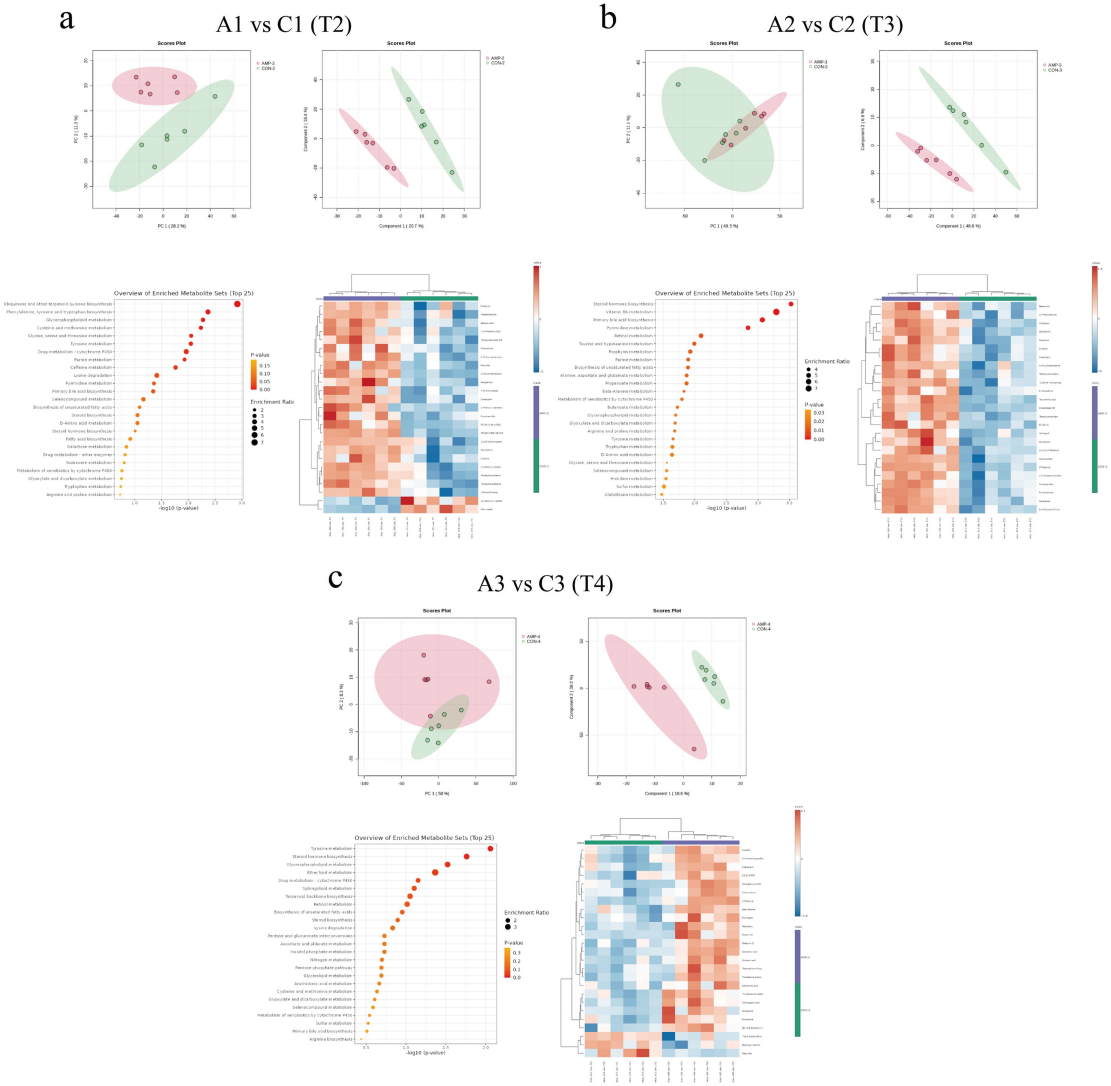
Effects of basal diet (CON) and antimicrobial peptides-supplemented diet (AMP) on serum metabolites in cats. **(a)** Serum metabolites prior to transportation [T2: end of pre-feeding period (before transportation)]; **(b)** Serum metabolites after transportation (T3: after transportation); **(c)** Serum metabolites after the recovery period (T4: end of recovery period). The panels include principal component analysis (PCA), partial least squares discriminant analysis (PLS-DA), the top 25 differentially expressed metabolites, and KEGG pathway enrichment bubble plots. Red and blue boxes represent positive and negative correlations.

As shown in Figure 9, PCA indicated a trend of separation between the two groups at T2, whereas no clear separation was observed at T3 and T4. In contrast, PLS-DA demonstrated distinct separations in serum metabolite profiles between the CON and AMP groups across all time points, suggesting the presence of differentially expressed metabolites. Compared to the CON group, 63 differentially expressed serum metabolites were identified at T2 in the AMP group, with 48 upregulated and 15 downregulated. At T3, 230 differential metabolites were detected, with 206 upregulated and 24 downregulated in the AMP group. By T4, a total of 50 differential serum metabolites were identified between the two groups, among which 37 were significantly upregulated and 13 were significantly downregulated in the AMP group.

KEGG pathway enrichment analysis revealed that the differential metabolites were predominantly enriched in pathways related to amino acid metabolism (e.g., phenylalanine, tyrosine and tryptophan biosynthesis, tyrosine metabolism, cysteine and methionine metabolism), glycerophospholipid metabolism, purine metabolism, and arachidonic acid metabolism in the AMP group at T2 (Figure 9a). At T3, additionally affected pathways encompassed lipid metabolism (e.g., α-linolenic acid metabolism and primary bile acid biosynthesis), pyrimidine metabolism, steroid hormone biosynthesis, retinol metabolism, taurine and hypotaurine metabolism, as well as glyoxylate and dicarboxylate metabolism (Figure 9b). At T4, these differential metabolites were primarily associated with amino acid metabolism pathways (e.g., alanine, aspartate and glutamate metabolism, glycine, serine and threonine metabolism, and tyrosine metabolism), along with ascorbate and aldarate metabolism, arachidonic acid metabolism, glyoxylate and dicarboxylate metabolism, and steroid hormone biosynthesis (Figure 9c).

### 3.8 Correlation between differential metabolites and fecal bacteria at the genus level

**Figure 10:**
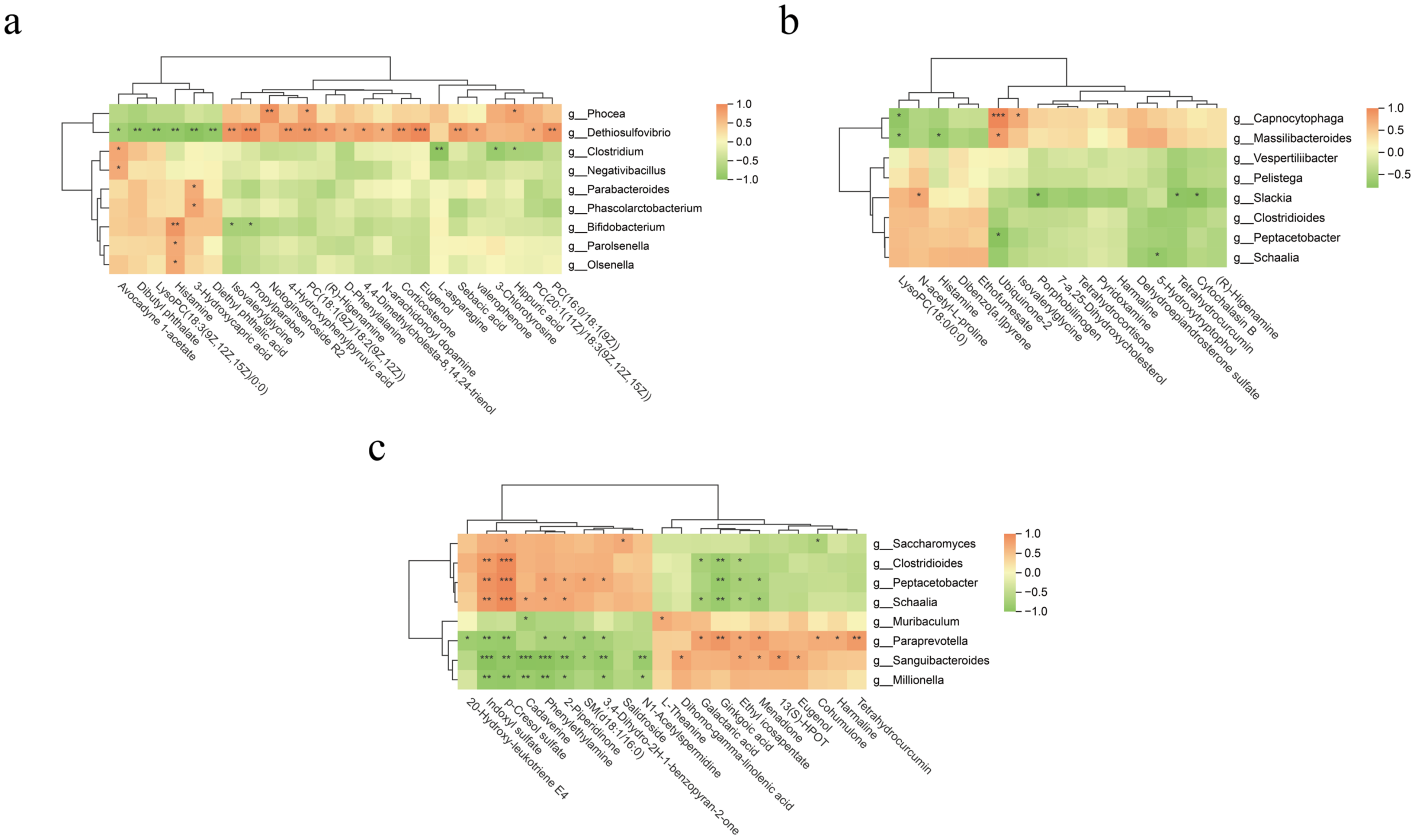
Spearman correlation analysis between the differential fecal metabolites and microbiota in CON groups and AMP groups prior to transportation [**a**; T2: end of pre-feeding period (before transportation)], after transportation (**b**; T3: after transportation), and after the recovery period (**c**; T4: end of recovery period). The symbol (*) indicates a significant correlation between metabolites and fecal bacteria (*p < 0.05, **p < 0.01, ***p < 0.001). Orange color indicates a positive correlation, and green color indicates a negative correlation.

Correlation between differential metabolites (fecal and serum) and fecal bacteria (at the genus level) is displayed in the heatmap (Figure 10). At T2, the genus *Phocea* exhibited significant positive correlations with hippuric acid, PC(20:1(11Z)/18:3(9Z,12Z,15Z)), and notoginsenoside R2. In contrast, *Dethiosulfovibrio* was positively correlated with corticosterone, 4,4-dimethylcholesta-8,14,24-trienol, 4-hydroxyphenylpyruvic acid, (R)-higenamine, and psilocin, while showing negative correlations with dibutyl phthalate, LysoPC(18:3(9Z,12Z,15Z)/0:0), histamine, and 3-hydroxycapric acid. *Clostridium* demonstrated negative correlations with 3-chlorotyrosine, hippuric acid, and L-asparagine, but a positive correlation with avocadyne 1-acetate (Figure 10a).

At T3, *Capnocytophaga* displayed positive associations with ubiquinone-2 and isovalerylglycine, and a negative correlation with LysoPC(18:0/0:0). *Slackia* correlated positively with N-acetyl-L-proline, but negatively with porphobilinogen, cytochalasin B, and tetrahydrocurcumin. Additionally, *Schaalia* was inversely correlated with 5-hydroxytryptophol (Figure 10b).

At T4, a more complex interaction pattern emerged. *Paraprevotella* was positively correlated with ethyl icosapentate, menadione, harmaline, and tetrahydrocurcumin, whereas it showed negative correlations with 2-piperidinone, indoxyl sulfate, p-cresol sulfate, SM(d18:1/16:0), and 3,4-dihydro-2H-1-benzopyran-2-one. Similarly, *Sanguibacteroides* exhibited positive correlations with dihomo-gamma-linolenic acid, ethyl icosapentate, 13(S)-HPOT, and menadione, but negative correlations with indoxyl sulfate, cadaverine, phenylethylamine, and p-cresol sulfate. Conversely, the three genera *Clostridioides*, *Peptacetobacter* and *Schaalia* were consistently positively correlated with p-cresol sulfate and indoxyl sulfate, negatively correlated with ginkgoic acid and ethyl icosapentate (Figure 10c).

## 4 Discussion

### 4.1 Effects of AMPs on body weight, average daily feed intake, and fecal scores

Our study found that AMPs significantly attenuated the decrease in ADFI induced by transportation stress during the post-transport period. However, no significant changes in body weight or fecal scores were observed in two groups, suggesting that the stress induction protocol did not elicit prolonged stress in cats.

### 4.2 Effects of AMPs on sleep, activity, and behavior in cats

In this study, AMPs supplementation significantly improved sleep quality and behavioral performance in cats subjected to transport stress. Poor sleep quality and behavioral disturbances are often associated with elevated stress and anxiety levels in animals (25). Previous studies have reported that elevated serum tryptophan levels and enhanced melatonin synthesis are correlated with improved sleep quality in elite athletes (26). In line with previous findings, AMPs treatment resulted in significant upregulation of tryptophan metabolism pathways, as indicated by metabolomic analysis and was associated with extended nocturnal sleep duration in cats. During and shortly after transportation, activity levels were significantly lower in the AMP group than in the CON group, indicating lower level of restlessness. This effect may also be linked to the upregulation of the tryptophan metabolism pathway observed in the AMP group, which is hypothesized to mitigate excessive activity and anxious behaviors under stress conditions (27). Furthermore, this pathway may modulate GABAergic neuronal activity (28), as activation of the central GABAergic neurons inhibits sympathetic nervous system, which might reduce the secretion of stress hormones such as cortisol, and alleviate the tension and restlessness associated with transport stress (29).

Cats experiencing acute stress typically exhibit behavioral changes compared to unstressed individuals, including reduced exploration and increased behaviors such as pacing and vocalization (21, 30). These behavioral alterations are widely recognized as effective indicators of stress in cats. In the first OFT, AMP group showed a marked reduction in escape-related behaviors and pacing, while also demonstrating increased exploratory behavior. In the second OFT conducted at the end of the recovery period, cats in the AMP group also displayed fewer escape attempts and reduced hiding time. These results suggested that AMPs supplementation may effectively mitigate acute stress induced by transportation and enhance adaptation to novel environments in cats to novel environments. Furthermore, AMPs supplementation effectively alleviates stress responses (i.e., reduced CSS) induced by transportation and unfamiliar environments in cats, particularly during the first 1–2 days after transportation. Over time, CSS scores in both groups exhibited a decreasing trend, indicating a gradual reduction in stress as the cats acclimatized to the new environment, consistent with previous findings by Kessler and Turner (31). However, the AMP group exhibited a more rapid recovery, suggesting that AMPs may facilitate faster adaptation to novel environments.

### 4.3 Effects of AMPs on hematological parameters in cats

Acute stress typically induces changes in the WBC, LYM, and NEU counts and proportions, depending on the nature and intensity of the stressor (32). Acute stress in mice leads to transient migration of NEU and LYM from the circulating blood to other critical organs, and the elevated Epinephrine (EPI) can promote the proliferation of NEU, while COR reduces LYM population (32). In the present study, a similar trend was observed. NEU levels increased in all cats immediately after transportation, but rapidly returned to baseline by the end of the recovery period in the AMP group, whereas they continued to rise in the CON group. This suggests that AMPs may facilitate more efficient immune self-regulation and recovery rather than merely suppress the immune response, which could be beneficial for the long-term health of the cats. Additionally, WBC in the CON group significantly increased by the end of the recovery period compared to post-transportation levels, potentially reflecting a persistent inflammatory response to stress in a novel environment (33). The RBC count and HGB concentration are indicators of hematopoietic function (34). Under stress conditions, these parameters generally rise due to heightened oxygen demand (35). In this study, however, both groups exhibited decreasing trends in RBC and HGB, especially after transportation and by the end of the recovery. Increased cortisol secretion under stress may cause sodium and water retention and the increase in blood volume, leading to the dilution of RBC and HGB (36). Moreover, previous studies have suggested that changes in RBC and HGB are more influenced by medications or other external factors than by stress itself, thus may not serve as reliable indicators of stress (37, 38).

### 4.4 Effects of AMPs on serum hormones, inflammatory cytokines, immunoglobulins, and antioxidant capacity in cats

As a neurotrophic factor, BDNF plays a critical role in neuronal survival and synaptic plasticity (39). Previous studies have indicated that BDNF levels tend to increase in response to stress; however, prolonged stress typically leads to its downregulation (40). A similar pattern was observed in the present study. We hypothesize that the elevated BDNF levels initially observed may be attributed to stress responses induced by frequent sampling. Notably, the higher levels of BDNF observed in the AMP group suggest that AMPs may exert neuroprotective effects during the course of the experiment, potentially counteracting the detrimental effects of stress.

The HPA axis plays a pivotal role in regulating stress responses. Chronic stress can lead to dysregulation of HPA axis activity, impair immune function, and increase susceptibility to chronic and metabolic diseases (41). Our previous study demonstrated that AMPs derived from chicken intestines and cultured using *Bacillus subtilis*, are capable of reducing COR levels following transport stress in Ragdoll cats (17). The present study corroborates these findings. Furthermore, while the levels of ACTH and CRH in the control group decreased by the end of the recovery period, COR levels continued to rise. Elevation of COR may disrupt the negative feedback regulation within the HPA axis in the CON group (42). In contrast, CRH levels in the AMP group returned to pre-stress baseline levels by the end of the recovery and were significantly lower than those in the CON group, suggesting improved adaptation to the novel environment in AMP-treated cats.

Meanwhile, AMP supplementation significantly alleviated stress-induced inflammatory and oxidative responses, while enhancing immune function. Specifically, AMP not only attenuated the elevation of SAA but also increased levels of Apo-A1 and anti-inflammatory factors, indicating its anti-inflammatory properties and ability to curb the progression of inflammatory responses (17, 43). In terms of antioxidant effects, the elevated activities of T-AOC, GSH-Px, and SOD, along with the reduced MDA level after the recovery period, collectively demonstrate that AMP may enhance the free radical-scavenging capacity by boosting antioxidant enzyme activity, thereby mitigating oxidative stress-induced damage (44, 45). Furthermore, significantly elevated serum immunoglobulin levels in the AMP group both post-transport and after recovery further support the beneficial role of AMP in modulating immune function. In summary, these findings suggest that AMP ameliorates excessive inflammation triggered by transport and novel environment stress, and improves the overall physiological state of cats under stress, likely through elevating immunoglobulins and anti-inflammatory factors, enhancing antioxidant capacity, and downregulating pro-inflammatory cytokine expression.

### 4.5 AMPs altered the gut microbiota composition of cats

Stress is known to alter the gut environment and microbial composition, leading to dysbiosis in animals (14, 46). Numerous animal studies have shown that dietary AMPs modulate gut microbiota composition by suppressing pathogenic bacteria while promoting beneficial bacteria, thereby regulating gut microecology and maintaining intestinal microbiome diversity (9–11). Consistent with previous studies, our research identified Firmicutes, Bacteroidetes, Actinobacteria, and Proteobacteria as the predominant phyla in the feline intestinal microbiota (47). At the genus level, the relative abundances of *Bacteroides* and *Prevotella* genera remained consistently higher in the AMP group than in the CON group throughout the trial. Both genera belong to the phylum *Bacteroidetes* and are considered beneficial bacteria involved in the fermentation of polysaccharides and the production of SCFAs such as succinate and acetate. These SCFAs exhibit anti-inflammatory properties and contribute to the maintenance of normal intestinal barrier function (48).

The relative abundance of *Schaalia*, *Clostridioides difficile*, and *Bacteroides* were significantly higher in the CON group than in the AMP group. Commonly found in the oral cavity, gastrointestinal tract, and urinary system, *Schaalia* has been associated with the occurrence of inflammatory responses and infectious diseases (49). *Clostridioides difficile* is one of the most serious acquired intestinal pathogens capable of causing diseases ranging from mild diarrhea to life-threatening pseudomembranous colitis (50). The genus *Bacteroides* comprises several opportunistic pathogenic species that can induce infections across multiple organ systems under immunocompromised conditions (51). These findings indicate that AMPs supplementation may contribute to the maintenance of gut health by promoting beneficial microbiota and suppressing potentially harmful microorganisms.

SCFAs resulting from the fermentation of carbohydrates and branched-chain amino acids by specific gut bacteria, contribute to intestinal health by serving as an energy source for colonocytes, enhancing epithelial barrier integrity, modulating immune responses, and regulating host metabolism as well as gut–brain signaling (52). In contrast, BCFAs are produced primarily from protein fermentation (53) and have been shown to influence lipid and glucose metabolism (54). It has also been suggested that BCFAs may indirectly affect immune function by modulating metabolic processes in the adipose tissue and liver (55). In the present study, acetic acid and propionic acid levels exhibited a decreasing trend in both groups, particularly in control cats over time. However, the abundance of *Bacteroides*, the primary acetate-producing bacteria did not decrease significantly, suggesting that stress may suppress the metabolic activity of *Bacteroides* without reducing its population. Notably, butyric acid levels were significantly higher in the CON group than in the AMP group throughout the study period, but decreased markedly after 1 week of housing in the new environment. Butyric acid, as the primary energy source for colonic epithelial cells, is critical for maintaining intestinal barrier function and mitigating inflammation (56). Numerous studies have reported that levels of butyric acid fluctuate substantially during gut dysbiosis or compromised health (57). The relative stability of butyric acid in the AMP group suggests that AMPs supplementation may help maintain intestinal homeostasis by supporting butyrate-producing microbiota communities. BCFAs have been shown to promote the expression of anti-inflammatory factors, thus exerting preventive effects on intestinal inflammation (55). Isobutyric acid levels increased significantly in the AMP group after the recovery phase, whereas valeric acid levels remained stable throughout the trial. In contrast, valeric acid in the CON group significantly declined after the recovery phase. Collectively, these findings suggest that AMPs supplementation may confer anti-stress and anti-inflammatory benefits by modulating the composition and metabolic function of the intestinal microbiota.

### 4.6 AMPs altered the metabolism of cats

Metabolomic studies related to psychiatric disorders have revealed that dysregulation of amino acid, lipid, and energy metabolism is closely associated with the onset and progression of stress (58). Our study found that compared to the CON group, the differential metabolites in the AMP group before and after transportation were primarily enriched in pathways related to amino acid metabolism and lipid metabolism. As a precursor of nitric oxide (NO), arginine modulates synaptic plasticity and is thereby involved in regulating stress responses to psychological stimuli (59). Proline also affects synaptic plasticity, potentially through GABAergic signaling (60). Alterations in steroid hormone biosynthesis pathways suggest that AMPs may further enhance stress adaption in cats by modulating the HPA endocrine axis (61). Additionally, serum metabolomic profiling before transportation supports the preventive effect of AMPs against stress. Specifically, AMPs supplementation upregulated tryptophan metabolism, which serves as a precursor for serotonin (5-HT) and plays a beneficial role in mood regulation, thereby contributing to improved sleep quality and reduced anxiety-related behavior (62). The upregulation of arachidonic acid metabolism pathway by AMPs might be another mechanism by which it exerts anti-inflammatory effects (63).

In addition to the aforementioned pathways, we observed that AMPs supplementation significantly upregulated several fecal metabolic pathways in cats after transportation, including tryptophan metabolism, α-linolenic acid metabolism, linoleic acid metabolism, biotin metabolism, and tyrosine metabolism. Linoleic acid has been shown to modulate astrocyte survival, GABAergic neurotransmission, and astrogenesis via the endocannabinoid system, thereby influencing anxiety and depressive-like behaviors (64). α-Linolenic acid, conversely, can enhance the secretion of BDNF, thereby promoting neurogenesis, neuronal cell survival, synaptic plasticity, and cognitive function, while reducing the risk of anxiety and depression (65). Furthermore, the serum metabolomic analysis revealed a significant upregulation of the bile acid metabolism pathway in the AMP group after transportation. Bile acid is not only integral to lipid digestion and absorption but also functions as signaling molecule that regulates multiple physiological processes, including energy metabolism and inflammatory responses (66). Previous studies indicate that bile acid confers beneficial effects on intestinal health by activating receptors such as the farnesoid X receptor (FXR) and the G protein-coupled bile acid receptor TGR5, which enhance intestinal barrier integrity, suppress pro-inflammatory cytokine production, and modulate gut microbiota composition (67).

After 1 week of housing in the new environment, compared to pre- and post-transportation periods, enriched metabolic pathways in the AMP group were related to amino acid metabolism, lipid metabolism, and other metabolic processes, suggesting a sustained regulatory effect of AMPs on feline metabolism. Additionally, a significant upregulation of the riboflavin metabolism pathway was observed in the fecal metabolome at the end of the recovery period. Riboflavin acts as an essential cofactor for multiple redox enzymes involved in electron transport and energy production (68). Modulation of this pathway by AMPs may support mitochondrial function and attenuate the oxidative damages induced by stress. Acetoacetate and dicarboxylic acid metabolism play crucial roles in the tricarboxylic acid (TCA) cycle and gluconeogenesis, and alterations in these pathways reflect shifts in cellular energy status (69). The observed changes in serum acetoacetate and dicarboxylic acid metabolism during both transportation and recovery phases imply that AMPs may facilitate host adaptation to stress through regulation of energy metabolism. As an important antioxidant, ascorbic acid (vitamin C) scavenges free radicals, and protects cells from oxidative damage (70). Upregulation of ascorbate and aldarate metabolism pathways in serum by the end of recovery suggests that AMPs also mitigate oxidative stress by reinforcing innate antioxidant capacity.

Based on the correlation analysis, it was observed that supplementation with the AMP formulation significantly modulates the composition of gut microbiota and metabolites in cats. These alterations are associated with enhanced anti-inflammatory and antioxidant capacity, maintenance of immune homeostasis, and consequently, improved stress resistance (71).

### 4.7 AMPs altered the transcriptomic profile of cats

The pre-transport upregulation of key pathways in the AMP group reveals a potential state of physiological pre-conditioning. Studies have shown that anterograde synaptic transmission can modulate synaptic transmission and stabilize inhibitory synapses by activating postsynaptic receptors (e.g., GABA receptors), thereby influencing the excitatory-inhibitory balance in neural circuits (72). Genes involved in this signaling pathway are strongly associated with psychiatric disorders such as autism, and dysregulation of this process may contribute to the pathophysiology of certain mental health conditions (73). Protein activation cascades are core components of various signaling pathways, and their upregulation suggests enhanced intercellular communication and signal transduction, which may facilitate a more coordinated response to stress (74). Protease-activated cascades, such as the MAPK cascade, play crucial roles in mediating cellular responses to environmental challenges and moderate upregulation of these pathways has been shown to improve cellular resilience to oxidative stress (75). The biosynthesis of pantothenic acid and coenzyme A may support stress adaptation by modulating ATP synthesis and alleviating oxidative stress (76). As an essential cofactor, coenzyme A is integral to the tricarboxylic acid (TCA) cycle and fatty acid metabolism, the upregulation of which may promote more efficient mobilization of energy reserves during stress responses in cats (77).

Post-transport analysis revealed that AMP supplementation enhanced immunocompetence and tissue repair mechanisms, while attenuating excessive inflammation. Specifically, the complement system is critical in the clearance of damaged cells and potential pathogens, which can be particularly protective against the immune dysfunction induced by stress (78). The significant upregulation of both classical and alternative complement activation pathways suggests that AMPs may enhance immune competence. The Hippo signaling pathway has been shown to play a central role in tissue regeneration and the regulation of organ size, potentially by influencing cell proliferation (79). The activation of Hippo pathway following stress may facilitate the repair of tissue damage, and the upregulation of this pathway by AMPs also indicated its protective effects in reducing post-stress tissue damage in cats. Previous studies have demonstrated that excessive activation of the TLR signaling pathway is closely associated with dysregulated inflammatory responses (80). In our study, a significant downregulation of the TLR signaling pathway was observed in the AMP group after transportation. This finding is consistent with earlier reports suggesting that AMPs can suppress TLR-mediated signaling pathway, thereby attenuating excessive inflammation (81). The observed downregulation of this pathway further supports the protective role of AMPs in mitigating hyper-inflammatory responses and preventing the persistent inflammatory and immunosuppressive states often associated with chronic stress.

Following 1 week of acclimation in the new environment, transcriptional profiling indicated that AMP supplementation enhanced cellular responsiveness and promoted metabolic and neurological recovery. Cell signaling is a key mechanism whereby cells perceive and respond to external stimuli such as chemical signals, mechanical stress, and temperature variations (82). Different cell signaling pathways regulate nearly all aspects of cellular activity, including metabolism, motility, and proliferation and differentiation (83). The upregulation of cell signaling implies enhanced cellular perception and response mechanisms to external stimuli, which may consequentially modulate lipid metabolism, energy homeostasis, and many other biological processes (84). Fatty acid derivatives, including lipoxins, prostaglandins, and endocannabinoids, play critical roles in inflammation, neuroprotection, and mood regulation (85, 86). These derivatives, which are derived from polyunsaturated fatty acids, exhibit potent anti-inflammatory and tissue reparative effects (87). The upregulation of the retrograde endocannabinoids signaling pathway, identified in the KEGG analysis, further supports this observation. Endocannabinoids are known to participate in stress recovery and mood regulation and the upregulation of this pathway may therefore promote emotional stability and facilitate physiological recovery following stress (88). Previous studies have confirmed that activation of the endocannabinoid system can mitigate the adverse effects of transportation stress in cats (89), which is consistent with our findings. Therefore, we propose that AMPs may alleviate stress caused by unfamiliar environments in cats by modulating metabolic and inflammatory responses and upregulating retrograde endocannabinoid signaling pathways.

## 5 Conclusion

In summary, this study systematically evaluated the effects of AMPs supplementation on transportation- and novel environment-induced stress in cats. Both groups maintained stable body weight and normal fecal throughout the experimental period. Although transportation stress significantly suppressed ADFI, AMPs supplementation markedly improved ADFI in the AMP group. AMPs supplementation also improved nocturnal sleep and daytime rest following transportation, while mitigating the sharp decline in physical activity. The CSS was significantly lower in the AMP group than in the CON group on day 1–3 after transportation. In the OFT, the AMP group exhibited significantly reduced hiding and escape behaviors compared to the CON group. Furthermore, AMPs supplementation significantly decreased the levels of HPA axis hormones such as COR and CRH, suppressed pro-inflammatory cytokines including IL-1β, IL-6, and SAA, and increased anti-inflammatory and immune indicators such as IL-10, IgG, and Apo-A1, demonstrating its immunomodulatory and anti-inflammatory potential. Additionally, AMPs enhanced the activity of antioxidant enzymes (GSH-Px, SOD, and CAT), while reducing MDA levels, thereby alleviating transportation-induced oxidative stress. Gut microbiota analysis revealed that AMPs supplementation decreased the relative abundance of pathogenic genera such as *Shaalia* and *Actinomyces*, while enriching beneficial genera including *Parabacteroides* and *Paraprevotella*, thus contributing to improved intestinal homeostasis. Metabolomic profiling indicated that AMPs enhanced amino acid, lipid, and energy metabolism pathways, which in turn attenuated oxidative damage and inflammatory responses, supporting systemic health under transportation-induced stress. Transcriptomic analysis further demonstrated that AMPs upregulated signaling pathways associated with complement activation, lipid metabolism, and cellular signal transduction, while downregulating virus-related processes and pathways associated with cellular damage. These coordinated molecular changes by AMPs treatment collectively promoted stress alleviation and facilitated physiological recovery.

## Data Availability

The original contributions presented in the study are publicly available. Both metagenomic data and transcriptomic data are deposited in the NCBI repository, with accession numbers PRJNA1402009 and PRJNA1405318 respectively. The metabolomics data have been deposited to MetaboLights repository with the study identifier MTBLS13746, link: https://www.ebi.ac.uk/metabolights/index.

## Footnotes

1
https://www.metaboanalyst.ca/
