# Supplementary material for: Effects of an antimicrobial peptide on transport- and novel environment-induced stress in British Shorthair cats
Source: Front Vet Sci. 2026 Feb 5;12:1724637. doi: 10.3389/fvets.2025.1724637 (PMC12917902; doi:10.3389/fvets.2025.1724637)
Supplement: Supplementary file 1 [file Presentation_1.ZIP › supplementary figure/Figure legend.docx]

**Supplementary Figure 1 |** Effects of basal diet (CON) and antimicrobial peptides-supplemented diet (AMP) on feed intake, fecal score, and body weight in cats. (a) Feed intake; (b) Body weight; (c) Fecal score. Data are presented as the mean ± SEM. Different lowercase letters indicate statistically significant differences (*P* < 0.05), while the symbol (#) denotes a trend toward significance (*P* < 0.10). T1: End of diet transition period; T2: End of pre-feeding period (before transportation); T3: After transportation; T4: End of recovery period.窗体顶端窗体底端

**Supplementary Figure 2 |** Effects of basal diet (CON) and antimicrobial peptides-supplemented diet (AMP) on sleep time duration (a-b) and activity levels (c-g) in the cats. (a) Nocturnal sleep duration; (b) Total daytime microsleep duration; (c) Total daily activity; (d) Activity during transportation; (e) Activity within 1 hour post-transportation; (f) Activity within 4 hours post-transportation; (g) Activity within 1–3 hours before and after transportation. Data are presented as the mean ± SEM. Different lowercase letters indicate statistically significant differences (*P* < 0.05), while the symbol (#) indicates a trend toward significance (*P* < 0.10). BT, right before transportation; AT, right after transportation; AR: right after recovery.

**Supplementary Figure 3 |** Effects of basal diet (CON) and antimicrobial peptides-supplemented diet (AMP) on blood cell in the cats. (a) White blood cells, WBC; (b) Neutrophils, NEU; (c) Lymphocytes, LYM; (d) Red blood cells, RBC; (e) Hemoglobin concentration, HGB. Red lines in the figures indicate the reference ranges for each parameter. Data are presented as the mean ± SEM. Different lowercase letters indicate statistically significant differences (*P* < 0.05), while the symbol (#) indicates a trend toward significance (*P* < 0.10). T1: End of diet transition period; T2: End of pre-feeding period (before transportation); T3: After transportation; T4: End of recovery period.

**Supplementary Figure 4 |** Effects of basal diet (CON) and antimicrobial peptides-supplemented diet (AMP) on fecal SCFAs (a-c) and BCFAs (e-g) in the cats. (a) Acetate; (b) Propionate; (c) Butyrate; (d) Isobutyric acid; (e) Isovaleric acid; (f) Valeric acid. Data are presented as the mean ± SEM. Different lowercase letters indicate statistically significant differences (P < 0.05), while the symbol (#) indicates a trend toward significance (P < 0.10). T2: End of pre-feeding period (before transportation); T3: After transportation; T4: End of recovery period.

**Supplementary Figure 5 |** Effects of basal diet (CON) and antimicrobial peptides-supplemented diet (AMP) on α-diversity and beta (PCoA). (a) Shannon index; (b) Simpson index; (c) Chao1 index; (d) Goods coverage index; (e) Principal coordinate analysis (PCoA) plot. Data are presented as the mean ± SEM. Different lowercase letters indicate statistically significant differences (*P* < 0.05), while the symbol (#) indicates a trend toward significance (*P* < 0.10). T2: End of pre-feeding period (before transportation); T3: After transportation; T4: End of recovery period.

**Supplementary Figure 6 |** Venn diagram (a) and results of differentially expressed genes (DEGs, b). A1 vs C1, End of diet transition period (T1); A2 vs C2, End of pre-feeding period (T2); A3 vs C3, After transportation (T3); A4 vs C4, End of recovery period (T4).

**Supplementary Figure 7 |** KEGG enrichment analysis of differentially expressed genes (DEGs) at various time points. (a) A1 vs C1: End of diet transition period (T1); (b) A2 vs C2: End of pre-feeding period (T2); (c) A3 vs C3: After transportation (T3); (d) A4 vs C4: End of recovery period (T4).
