# Supplementary figures and images for: Effects of an antimicrobial peptide on transport- and novel environment-induced stress in British Shorthair cats
Source: Front Vet Sci. 2026 Feb 5;12:1724637. doi: 10.3389/fvets.2025.1724637 (PMC12917902; doi:10.3389/fvets.2025.1724637)

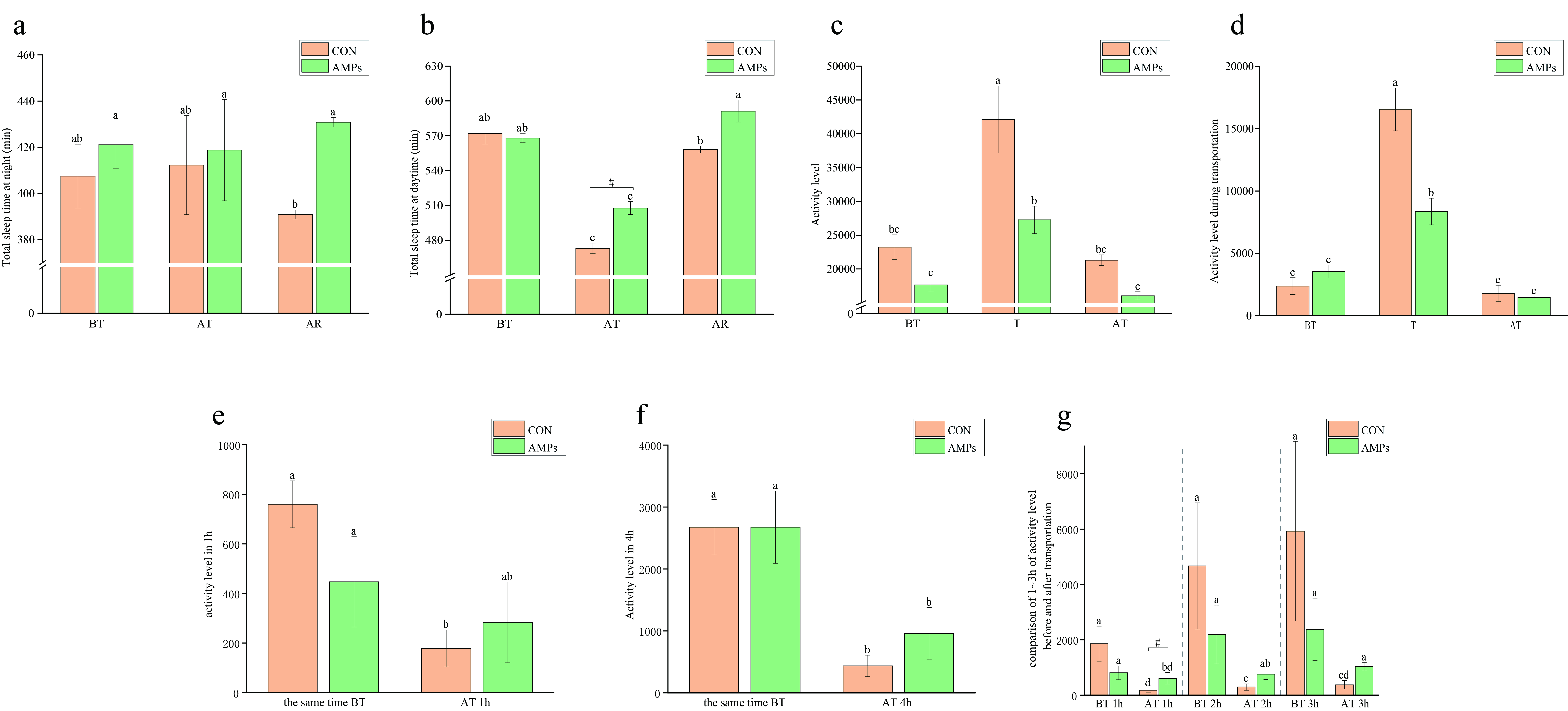

Supplement: Supplementary file 1 [file Presentation_1.ZIP › supplementary figure/supplementary figure 2.tif]

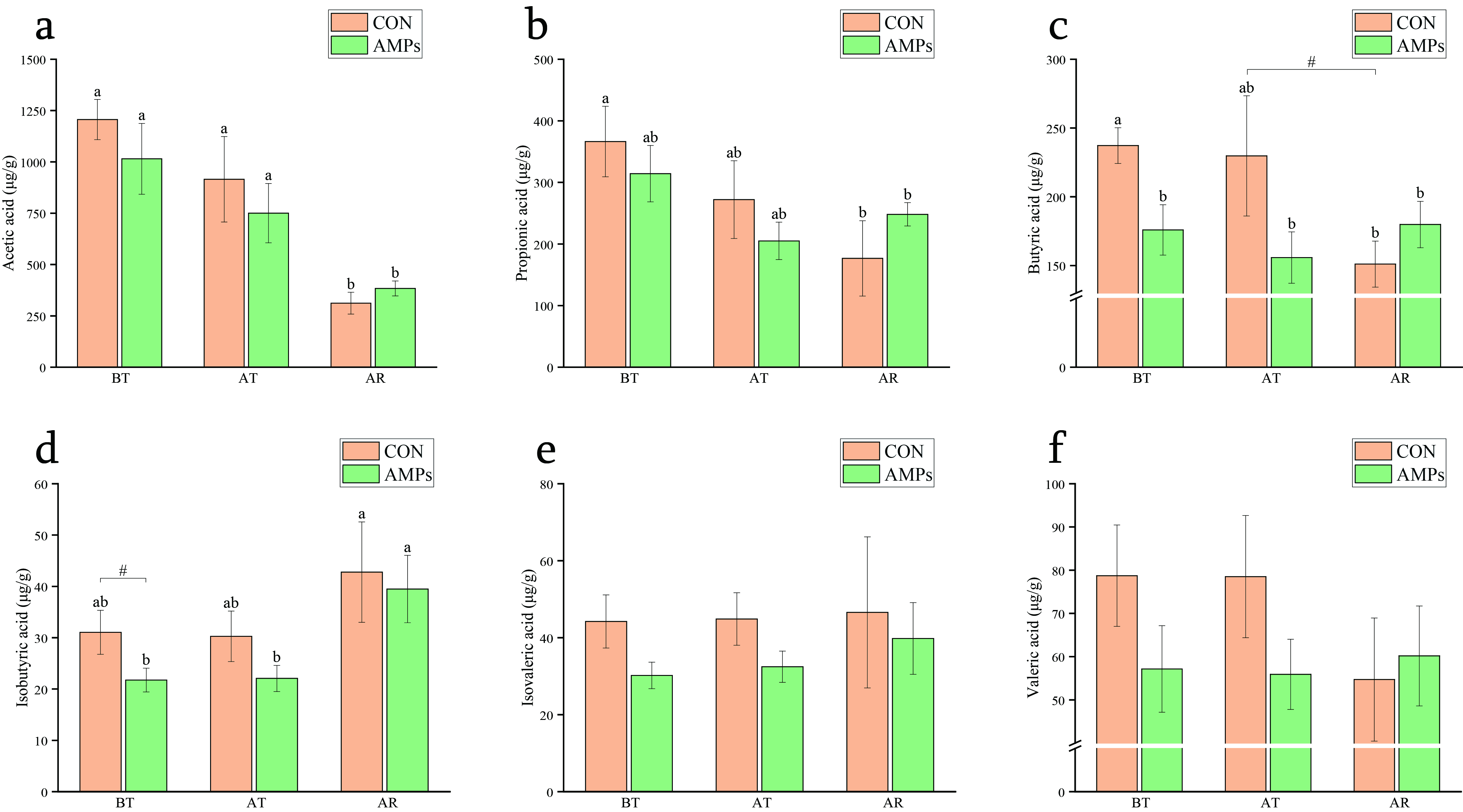

Supplement: Supplementary file 1 [file Presentation_1.ZIP › supplementary figure/supplementary figure 4.tif]

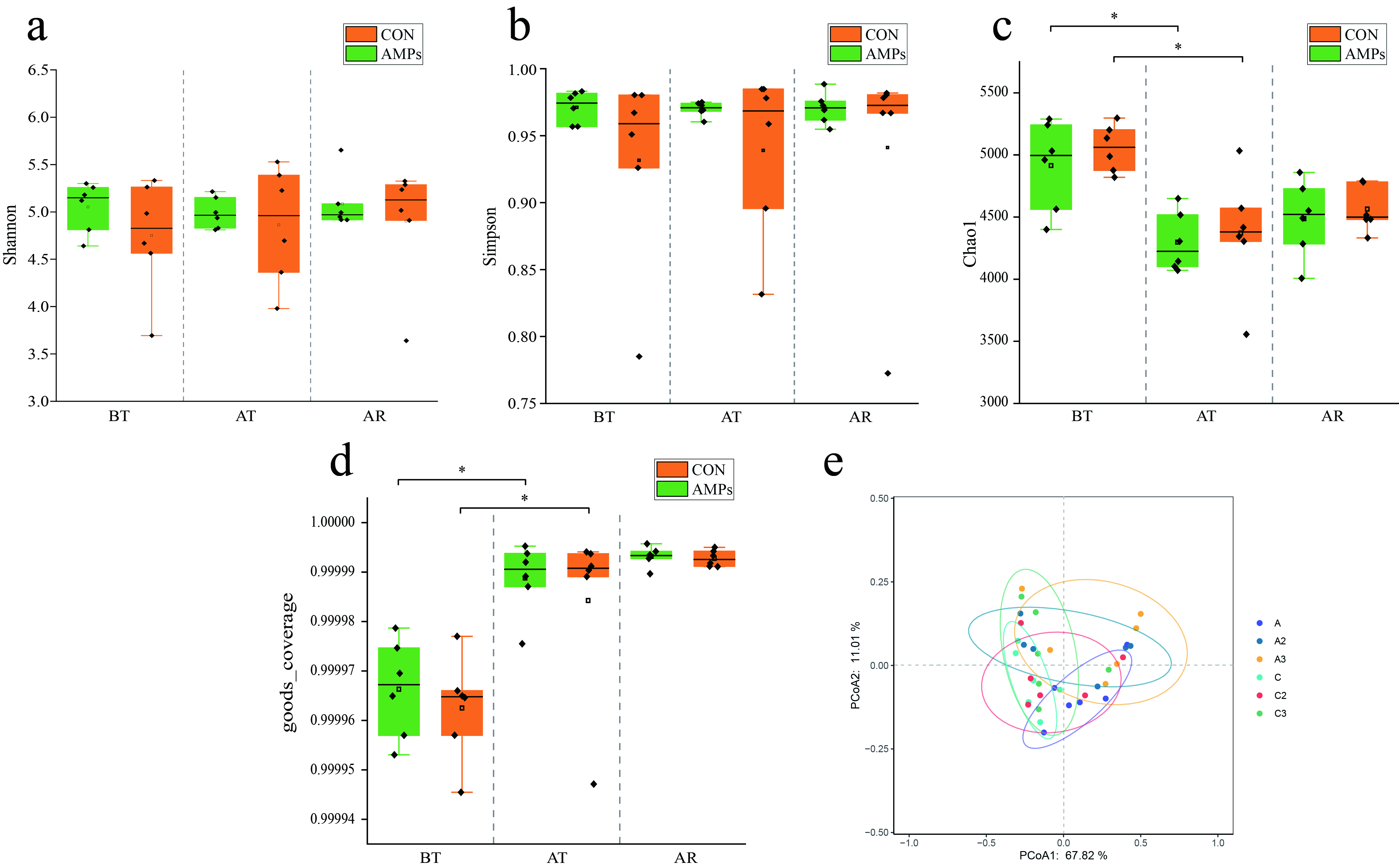

Supplement: Supplementary file 1 [file Presentation_1.ZIP › supplementary figure/supplementary figure 5.tif]

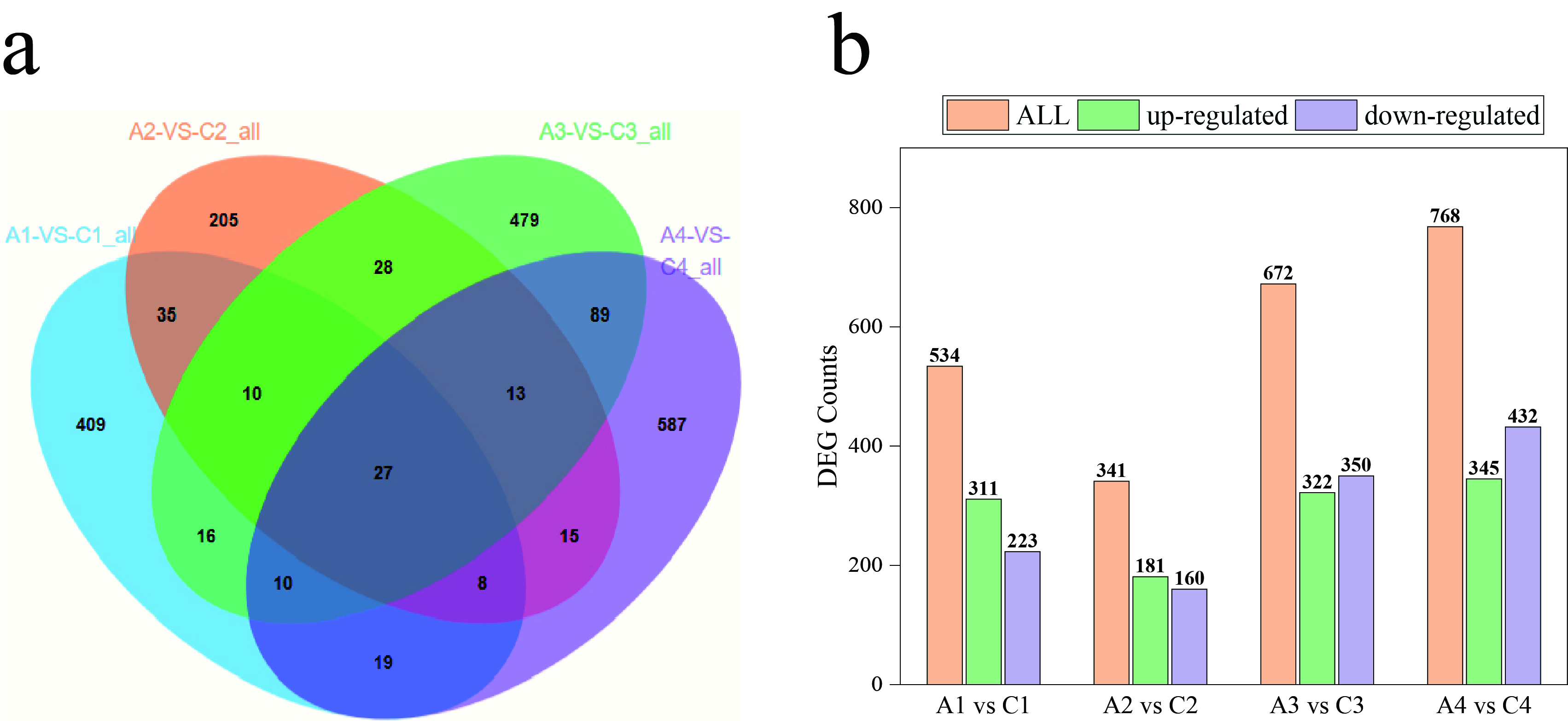

Supplement: Supplementary file 1 [file Presentation_1.ZIP › supplementary figure/supplementary figure 6.tif]

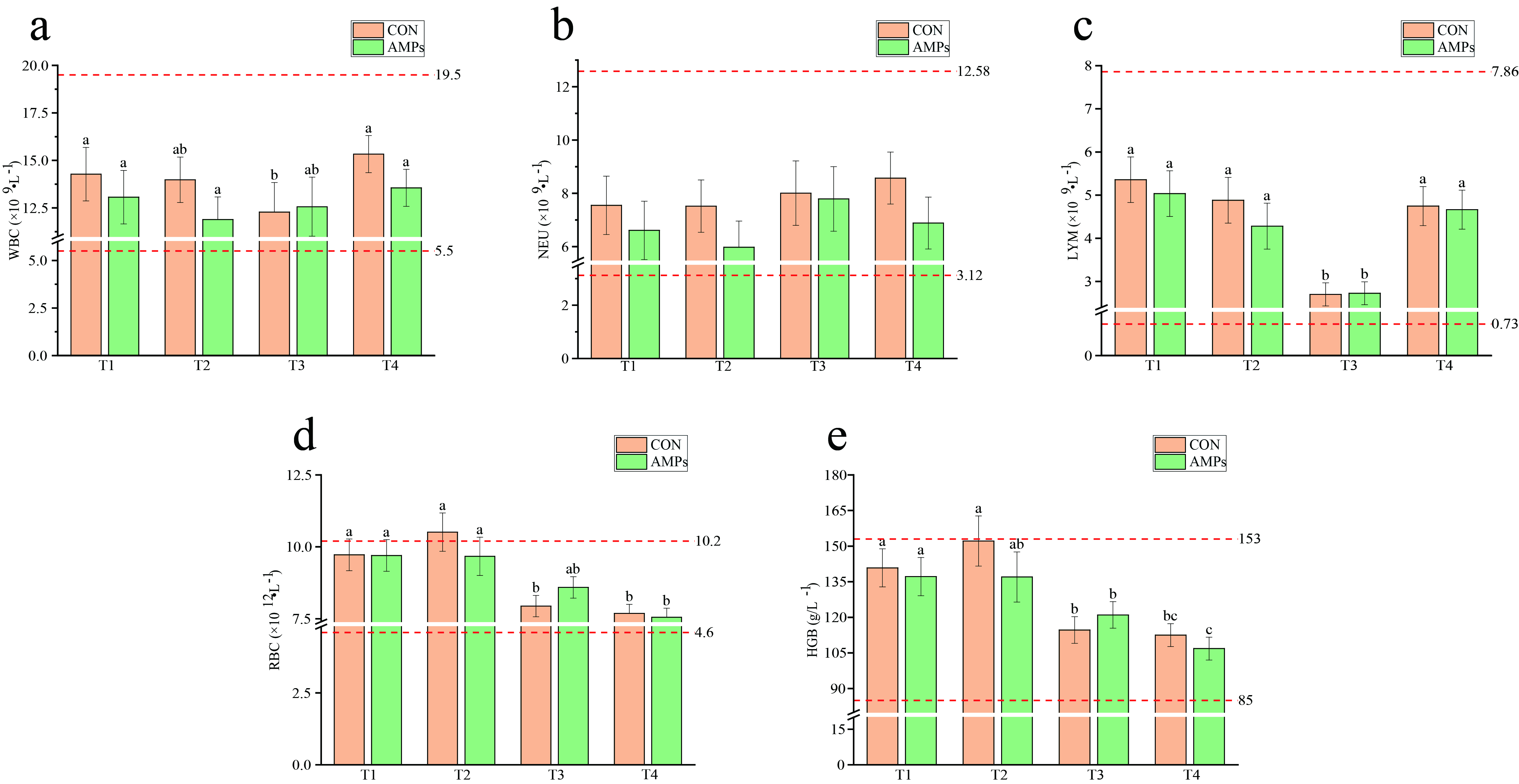

Supplement: Supplementary file 1 [file Presentation_1.ZIP › supplementary figure/supplementary figure 3.tif]

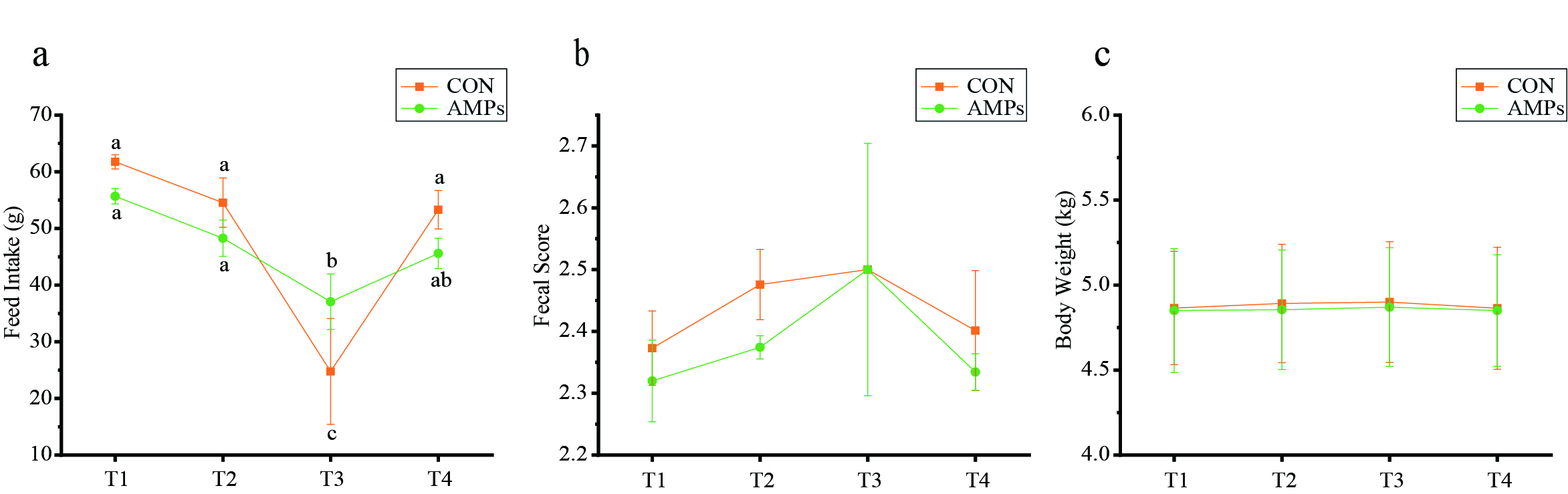

Supplement: Supplementary file 1 [file Presentation_1.ZIP › supplementary figure/supplementary figure 1.tif]

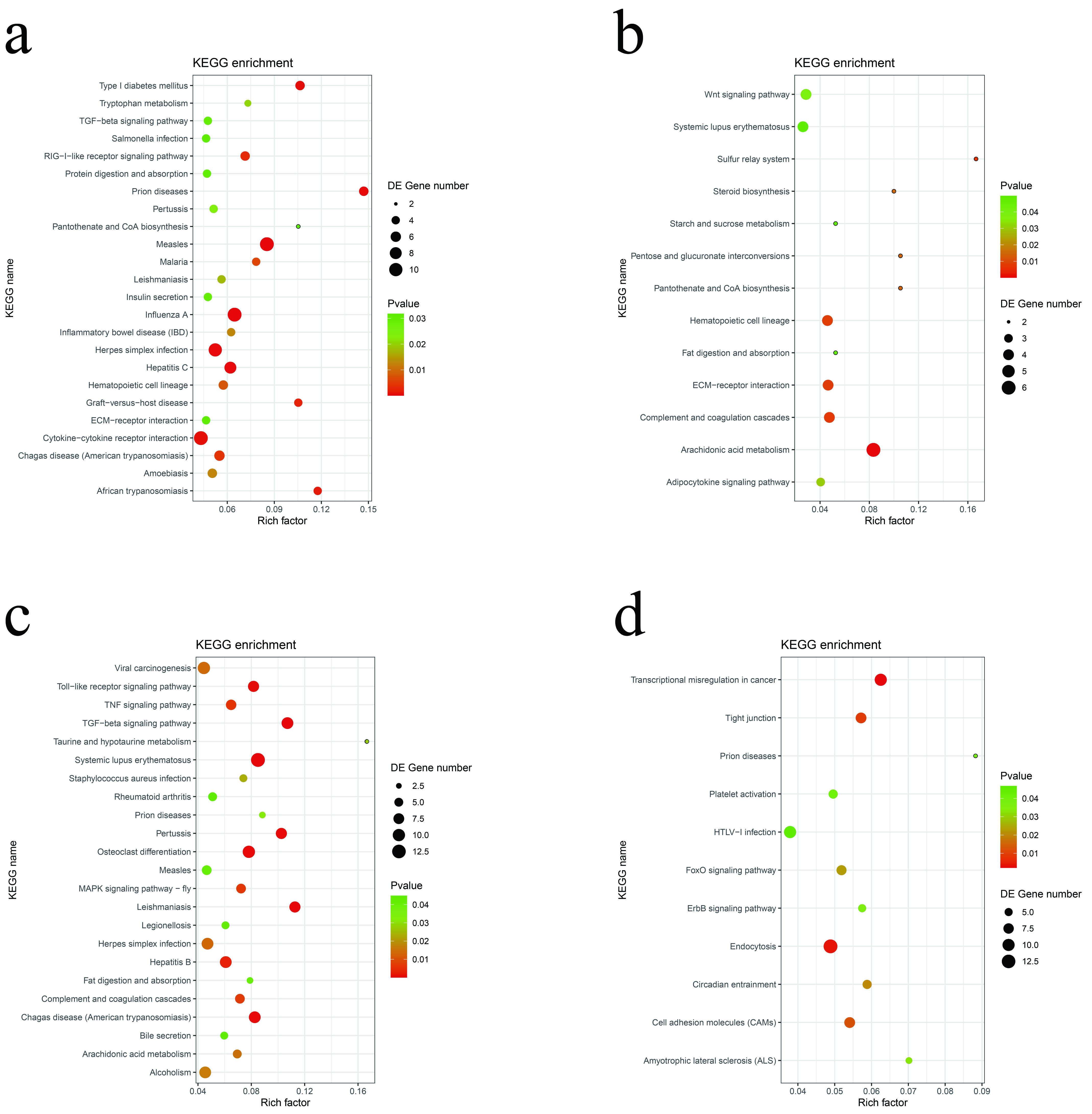

Supplement: Supplementary file 1 [file Presentation_1.ZIP › supplementary figure/supplementary figure 7.tif]
